# Phylogenomics reveals multiple evolutionary lineages of marine crustacean-infecting gregarine apicomplexans

**DOI:** 10.1038/s41598-026-46824-z

**Published:** 2026-04-20

**Authors:** Ina Na, Victoria K. L. Jacko-Reynolds, Corey C. Holt, Patrick J. Keeling

**Affiliations:** 1 Department of Botany, University of British Columbia, 3156-6270 University Blvd., Vancouver, BC V6T 1Z4 Canada; 2 Hakai Institute, Heriot Bay, BC Canada; 3 Department of Life Sciences, University of Bath, Bath, BA2 7AY UK

**Keywords:** Apicomplexa, Gregarines, Crustaceans, Cephaloidophoroidea, Phylogenomics

## Abstract

Apicomplexans are a diverse phylum of single-celled protists that are all obligate animal symbionts. The most well-studied members are severe pathogens of humans and livestock, such as the causative agents of malaria, cryptosporidiosis, toxoplasmosis, and coccidiosis, but comparatively little is known about their invertebrate-infecting counterparts, which are evolutionarily and ecologically important, but often overlooked. One of these groups is the Cephaloidophoroidea gregarine superfamily which is one of the most often-detected apicomplexan groups in molecular surveys of marine environments. The few members that have been isolated and molecularly characterized primarily infect crustaceans, and indeed all known crustacean-infecting gregarines phylogenetically branch within the Cephaloidophoroidea. Here we expand the phylogenomic data for gregarines from crustacean hosts, report 11 new species of crustacean-infecting gregarines, and perform phylogenomic analyses using transcriptome data to determine their position on the tree of Apicomplexa. The crustacean-infecting gregarines primarily do branch with the Cephaloidophoroidea, but not exclusively. *Lentusidium euphilomedae* n. gen. et sp., found in the seed shrimp *Euphilomedes* sp., branches very distantly to the Cephaloidophoroidea forming a strongly supported sister to Lecudinoidea. We show that crustacean-infecting gregarines comprise multiple evolutionary lineages providing important context for future interpretations of marine environmental surveys.

**Supplementary Information:**

The online version contains supplementary material available at 10.1038/s41598-026-46824-z.

## Introduction

Apicomplexans are one of the most widespread groups of microbial eukaryotic obligate symbionts, infecting a diverse range of terrestrial and aquatic vertebrate and invertebrate hosts^1,2^. The most well-studied apicomplexans are leading causes of life-threatening human diseases such as malaria, toxoplasmosis, and cryptosporidiosis (*Plasmodium* spp*.*, *Toxoplasma gondii*, *Cryptosporidium* spp., respectively)^3–5^, and deadly pathogens (*Babesia* spp., *Theileria* spp., and *Eimeria* spp.) of commercial livestock^6–8^, carrying major public health and economic consequences worldwide. Health and agricultural relevance have driven extensive research into this tiny proportion of apicomplexan diversity, leading to an incomplete and biased picture of their biology, diversity, and relevance to global ecosystems. Gregarines are a diverse and widespread apicomplexan group that predominantly infect the intestines of invertebrates^1^ including annelids^9–13^, insects^14–17^, bivalves^18^, echinoderms^19,20^, and crustaceans^21–23^. Features that distinguish gregarine apicomplexans include an epicellular feeding stage (clinically important apicomplexan parasites feed intracellularly)^24–28^, syzygy (a sexual reproduction stage in gregarines where trophozoites mature to male and female gamont cells that join in the host)^1^, reduction of mitochondrial and apicoplast machinery^17,29–33^, and distinct and diverse cell morphologies^13,34^. Efforts to fully understand gregarine diversity, phylogeny, and evolutionary history are ongoing, and have greatly benefitted from culture-free genomics such as single-cell transcriptomics. However, uneven sampling of lineages remains a challenge. High-throughput environmental sequencing surveys indicate that the abundance and diversity of gregarines dominate microeukaryotes in rainforest soil biomes^35^, and dominate apicomplexan diversity in marine environments^36^. Environmental surveys suggest that gregarines are much more diverse than previously thought, indicating a need for increased sampling efforts^37^. But the lack of phylogenetic representation limits the ability to interpret results from environmental surveys, highlighting the importance of characterizing neglected gregarine lineages.

In the marine environment, the Cephaloidophoroidea superfamily (such as Porosporidae, a family within the taxon)^23^ stands out as a devastatingly undersampled group that nevertheless dominates marine Apicomplexa surveys in relative abundance^2^. The Cephaloidophoroidea are the most highly represented Apicomplexa in open ocean, epipelagic, and benthic environments, as well as in mesoplankton symbiont communities^2,36^. Based on the few species that have been characterized directly, it is thought that Cephaloidophoroidea are highly host specific to crustaceans^21^, with the only known exception being *Cephaloidophora* cf. *flava* collected from a salp (*Thalicola flava*)^38^, and crustaceans are not infected by other gregarine taxa. Little is known about the biology and ecology of the Cephaloidophoroidea including the degree to which host fitness is impacted. Although environmental surveys strongly indicate Cephaloidophoroidea are globally abundant, they remain one of the most elusive and undersampled apicomplexans for molecular data. Genome data has been obtained from only four members of this superfamily. Other molecular data are available from only a handful of described species in the form of small and large subunit rDNA. The evolutionary relationships of members of the Cephaloidophoroidea, therefore, remain largely unclear with several instances of transferred species across genera^39^. The underrepresentation of this group also causes a problem where environmental sequences can be imprecise or misidentified due to a lack of characterized representatives in some databases. Many marine apicomplexan OTUs initially categorized as *Plasmodium* have since been revealed to be members of the Cephaloidophoroidea upon further scrutiny^2^.

To expand the known diversity of Cephaloidophoroidea molecular data, we isolated and generated single cell transcriptomes of several new crustacean-infecting gregarines (in addition to the previously described, *Ganymedes themistos*^40^) and determine the phylogenomic position of the newly discovered lineages. Here, we propose 11 new species isolated from crustacean mesobenthos and mesoplankton hosts. Morphological observations revealed notable diversity in cell shape, cell size, motility, and behavioural characters. We analyzed SSU/LSU rRNA and transcriptome data for phylogenetic and phylogenomic analyses and found all but one of the crustacean gregarines grouped in the superfamily Cephaloidophoroidea within the taxonomic family Cephaloidophoridae. Surprisingly, one crustacean gregarine branched outside the Cephaloidophoroidea, and instead branched with the Lecudinoidea, showing for the first time that crustacean-infecting gregarines are not restricted to the Cephaloidophoroidea.

## Results

### Morphology of new crustacean gregarine apicomplexan species and *Ganymedes themistos*

Eleven new gregarine species and one previously described species, *Ganymedes themistos,* were found in shrimp, juvenile crab larvae, barnacles, and amphipods in British Columbia, Canada (Supplementary Table S1, Supplementary Fig. S1). All isolated cells were either trophozoites or gamonts in syzygy. Detailed host, locality data, morphometrics, and morphology were recorded (Supplementary Table S1, S2).

**Fig. 1:**
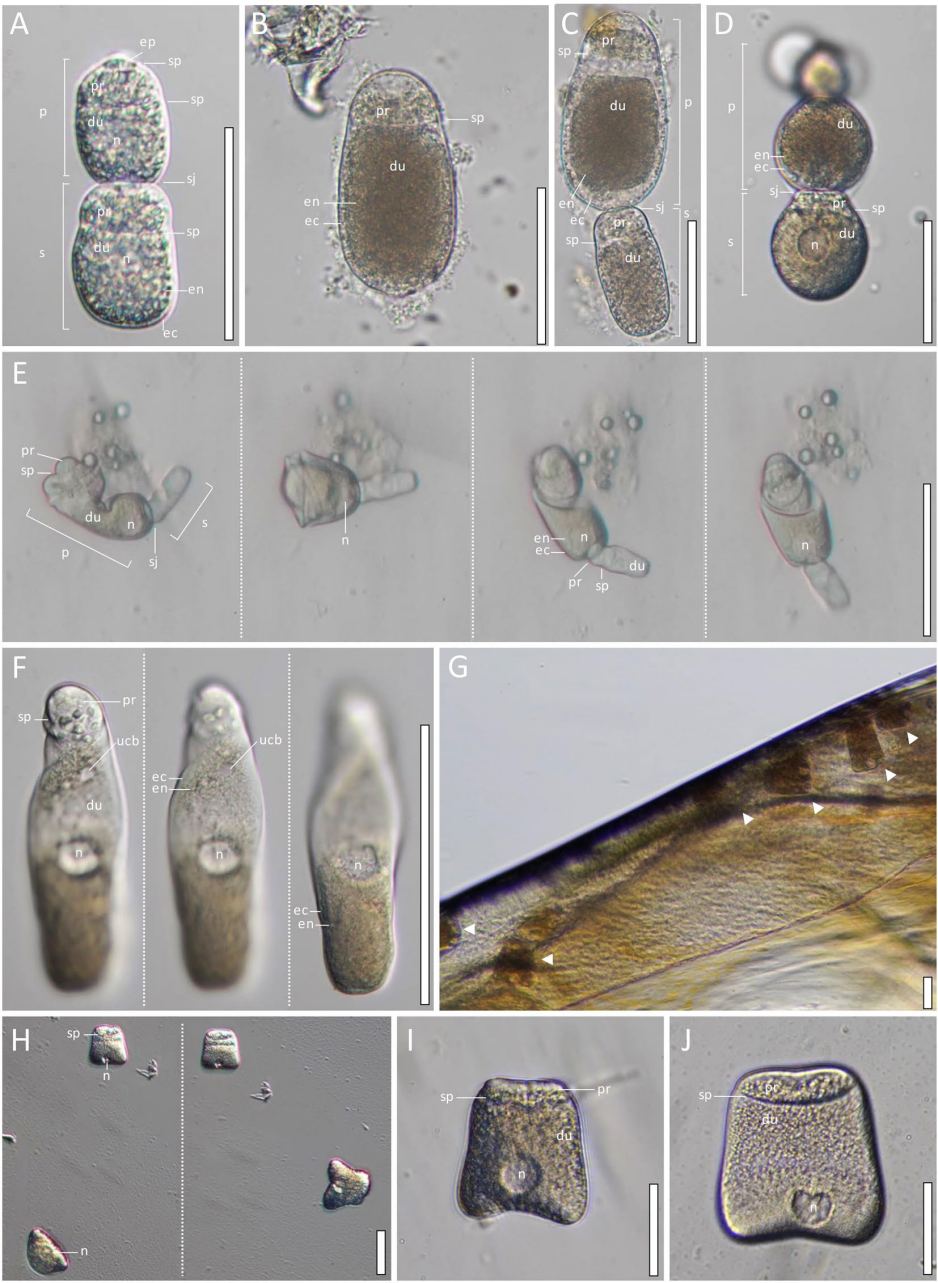
Light micrographs of new Cephaloidophoridae species showing the nucleus (n), ectoplasm (ec), endoplasm (en), unknown cell bodies (ucb), syzygy junction (sj) dividing the primite (p) and satellite (s), septa (sp) dividing the epimerite (ep), protomerite (pr), and deutomerite (du). (**A**) *Cephaloidophora hippola* n. sp. gamonts in caudo-frontal syzygy. (**B**) *Cephaloidophora caprellina* n. sp. trophozoite. (**C**) *Cephaloidophora caprellina* n. sp. gamonts in caudo-frontal syzygy. (**D**) *Cephaloidophora alienae* n. sp. gamonts in caudo-frontal syzygy. The primite was ruptured at the anterior. (**E**) *Cephaloidophora quadrae* n. sp. gamonts in caudo-frontal syzygy. Four frames of the gamonts as the cell exhibited gliding and contracting movement. (**F**) *Cephaloidophora cola* n. sp. trophozoite. (**G**) *Cephaloidophora squarepantsi* n. sp. (white triangle) visible through the body of the host. (**H**) *Cephaloidophora squarepantsi* n. sp. in two frames with the same two trophozoites in each frame. (**I**, **J**) *Cephaloidophora squarepantsi* n. sp. trophozoites. Scale bar: A, B, C, D, I, J = 50 µm. E = 30 µm. F, G, H = 100 µm.

*Cephaloidophora hippola* n. sp. gamonts (Fig. 1A) were collected from *Hippolyte* sp., a species of small, shallow-water inhabiting shrimp. The cells were capsule-shaped and approximately 33.5 µm in length and 22 µm in width. The primite and satellite pair were joined at a syzygy junction caudo-frontally where the posterior of the primite joins with the anterior of the satellite. Discreet septa separated the epimerite, protomerite, and deutomerite in the primite, and the protomerite and deutomerite in the satellite. A 11.5 µm diameter nucleus was located centrally in the deutomerite. A demarcation separated the ectoplasm and endoplasm.

A *Cephaloidophora caprellina* n. sp. trophozoite (Fig. 1B) and gamont pair (Fig. 1C) were collected from *Caprella* sp., a skeleton shrimp. The trophozoite and primite gamont were capsule-shaped, widening at the middle to posterior area and measured approximately 69 µm in length and 42 µm in width. The satellite gamont was bottle-shaped with flat lateral sides and rounded edges and measured 52 µm in length and 27 µm in width. A septum separated the protomerite and deutomerite. A nucleus could not be clearly seen through the darkly pigmented cytoplasmic material. The cells appeared to be translucent at the ectoplasm and in the outer edges of the endoplasm.

*Cephaloidophora alienae* n. sp. gamonts (Fig. 1D) were collected from opossum shrimp, *Alienacanthomysis macropsis*. The gamonts were spherical with a pointed anterior in the primite and flattened anterior with sharp edges at the syzygy junction in the satellite. The cells measured approximately 41 µm in length and 39.5 µm in width. The 13 µm diameter nucleus was spherical and located centrally in the cell. A discreet septum separated the protomerite and deutomerite of the satellite gamont. A distinction between ectoplasm and endoplasm was seen.

*Cephaloidophora quadrae* n. sp. gamonts (Fig. 1E) were collected from a *Caprella* sp. skeleton shrimp host. The primite was cylindrical and approximately 20 µm long and 10 µm wide. The satellite was significantly smaller at 11 µm in length and 5 µm in width. The spherical 6 µm diameter nucleus of the primite was located posteriorly and directly above the syzygy junction. A discreet septum separated the protomerite and deutomerite of the gamont. Ectoplasm and endoplasm separation was seen in the primite. The gregarine glided in the anterior direction while the primite exhibited repetitive bending movements: bending itself in half and twisting before slowly unfurling again (Supplementary Video 1).

*Cephaloidophora cola* n. sp. trophozoites (Fig. 1F) were collected from juvenile crab larval stages of the mud crab *Eriocheir sinensis*. Trophozoites were elongated and bottle-shaped measuring approximately 101 µm in length and 23.5 µm in width, which narrowed to 35 µm at the anterior and 38 µm at the posterior. The spherical nucleus was 11 µm in diameter and located centrally. The anterior of the cell had a rounded tip that indented at a discreet septum. A small distinct rounded body was observed in the region between the anterior and middle of the cell. Partitioning of the ectoplasm and endoplasm was seen. Slight undulating movements at the anterior portion of the cell was documented (Supplementary Video 2), otherwise no other movement was observed.

*Cephaloidophora squarepantsi* n. sp. trophozoites were collected from a species of opossum shrimp, *Neomysis* sp. where gregarines were visible through the translucent body of the host (Fig. 1G–J). The cells were approximately 89.3 µm long and 81.3 µm wide. The trophozoites resembled a rectangular prism with flat sides and rounded edges. In some cells the anterior was rounded (Fig. 1G), and in others the lateral sides and posterior were indented (Fig. 1H–J). Manually moved trophozoites showed that the cells were flat (20 µm width) with a bulging posterior (Fig. 1H). A septum separates the protomerite, and deutomerite. The spherical 23 µm diameter nucleus was located at the posterior of the cell.

**Fig. 2:**
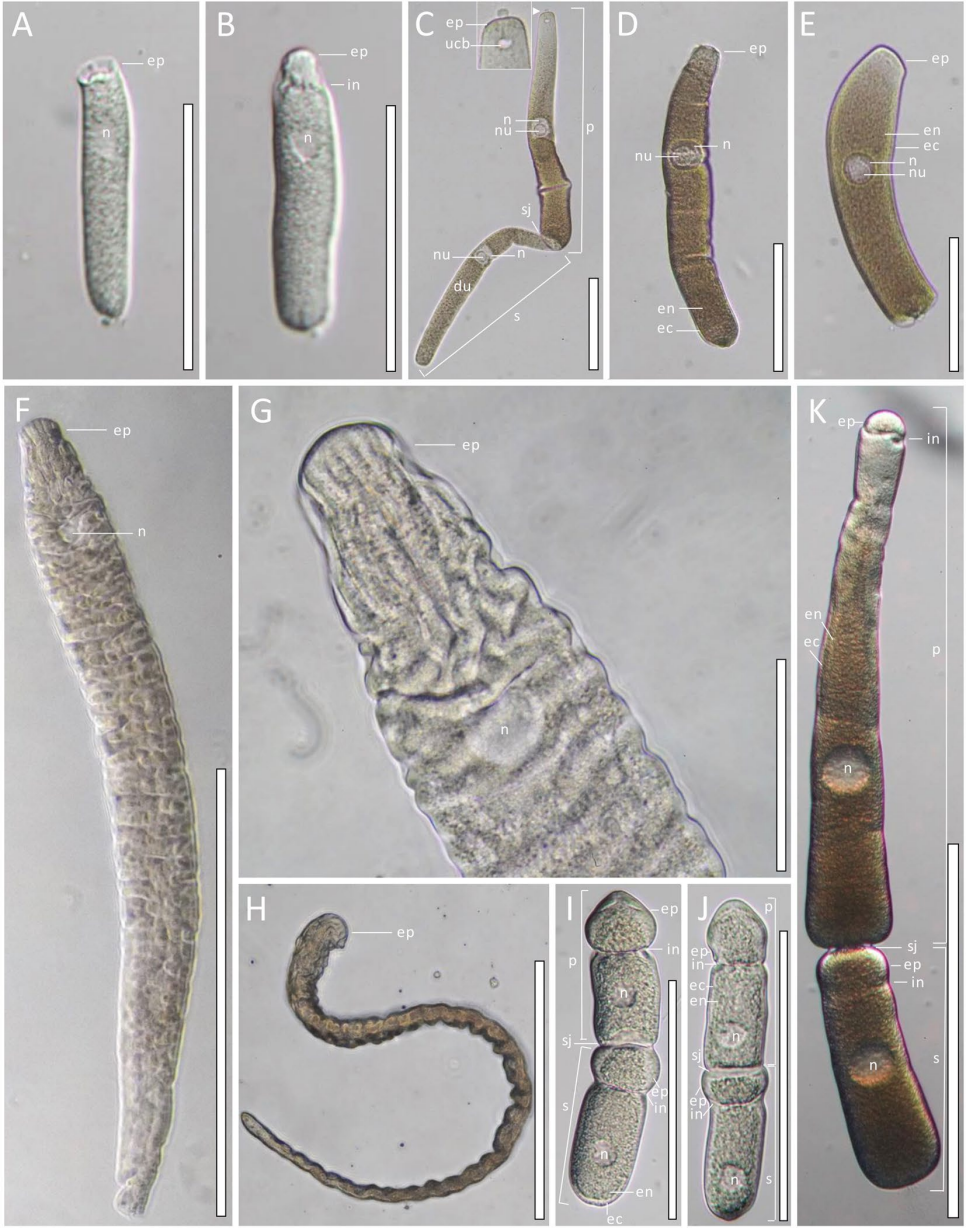
Light micrographs of Thiriotiidae and Ganymedidae showing the nucleus (n), nucleolus (nu), endoplasm (en), ectoplasm (ec), indentation (in) below the epimerite (ep), syzygy junction (sj) dividing the primite (p) and satellite (s), and unknown cell bodies (ucb). (**A**, **B**) *Thiriotia ampithae* n. sp. trophozoites. (**C**) *Thiriotia cypherae* n. sp. gamonts in caudo-frontal syzygy. (**D, E**) *Thiriotia cypherae* n. sp. trophozoite. (**F**) *Thiriotia zilla* n. sp. trophozoite. (**G**) Apical region of a *Thiriotia zilla* n. sp. trophozoite (**H**) *Thiriotia zilla* n. sp. trophozoite. (**I**, **J**) *Ganymedes balani* n. sp. gamonts in caudo-frontal syzygy. (**K**) A pair of *Ganymedes themistos* gamonts in caudo-frontal syzygy. Scale bar: A, B = 50 µm. C, D, E, G, I, J = 100 µm. F = 1000 µm. H = 500 µm. K = 300 µm.

*Thiriotia ampithae* n. sp. trophozoites (Fig. 2A,B) were obtained from the amphipod *Ampithoe valida*. The cells were cylindrical, 53.5 µm in length, and 10 µm in width with a rounded posterior. The anterior of other cells were smaller than the remainder of the cell body with an indentation between the two cell regions (Fig. 2B). The nucleus was 13 µm in diameter and situated in the region between the anterior and central area of the cell.

*Thiriotia cypherae* n. sp. gamont pairs and trophozoites (Fig. 2C–E) were collected from the amphipod *Cyphocaris challengeri*. The trophozoites and gamonts had varied morphology but were overall cylindrical with blunt ends and rounded edges. The gamonts were approximately 242 µm in length and 38.5 µm in width (Fig. 2C). In one cell a circular unknown structure was observed at the cell anterior. The trophozoites were approximately 227 µm in length and 37.5 µm in width (Fig. 2D,E). Some *T. cypherae* n. sp. trophozoites were slightly curved in resembling a crescent (Fig. 2E). The 22 µm diameter nucleus was spherical and centrally located. A clear demarcation separated the ectoplasm and endoplasm in both trophozoites. Creases could be seen on some cells (Fig. 2D). Others had a crown-like structure at the anterior of the cell which may be the result of disrupted syzygy.

*Thiriotia zilla* n. sp. trophozoites (Fig. 2F–H) were collected from juvenile *Scyra* sp*.*, a species of sharp-nosed crab. The trophozoite was vermiform with rounded ends at the anterior and posterior of the cell and measured 1844 µm in length and 154 µm in width. The nucleus was 48 µm in diameter and located at the anterior of the cell. The cell surface was folded into ridges, crossed vertically and horizontally, appearing lobulated in texture. The cell was also observed twisting over onto one edge, showing that the trophozoite was flatter on one pane (49 µm in width), reminiscent of *Cephaloidiophora squarepantsi* n. sp. trophozoites. Notably, trophozoites were able to bend, twist, and curl themselves forming circular, “C”, or “S” shapes while moving and exhibited quick gliding motion in the anterior direction (Supplementary Video 3, 4). The curling and twisting motion resembled the bending motility observed in distantly related archigregarines^11^.

*Ganymedes balani* n. sp. gamonts (Fig. 2I,J) were obtained from an acorn barnacle, *Balanus glandula*. Two similar morphologies were observed and processed as individual samples. Both exhibited enlarged epimerites forming an onion dome shape with a septum-like indentation that was narrower than the width of the cylindrical cell body. Type A gamonts (Fig. 2I) were approximately 65.5 µm in length and 26 µm in width. An overlapping region occurred between the tip of the satellite epimerite and posterior end of the primite. At the tip of the primite’s epimerite is a structure surrounding a canal leading into the cell (Fig. 2I). The 12 µm diameter nucleus was located centrally in the primite and in the middle to posterior area of the satellite. Type B gamonts (Fig. 2J) were approximately 51.3 µm in length and 20.3 µm in width. An overlap was not seen between the primite and satellite at the syzygy junction. The 9 µm diameter nucleus was located in the posterior region of the cell. Type A and B gamonts had an obvious ectoplasm and endoplasm.

*Ganymedes themistos* gamonts in syzygy (Fig. 2K) were found in the amphipod *Themisto libellula*. The primite measured 370 µm in length and 50 µm in width. The satellite was significantly shorter measuring 168 µm in length and approximately 56 µm in width. Below the epimerite was a thin septum-like indentation. The cells were cylindrical and widened towards the posterior. The rounded epimerite exhibited contracting movement (Supplementary Video 5). A septum-like indentation was below the epimerite. The spherical nucleus was 12 µm diameters and located in the central and central to posterior region of the cell. A separation between the ectoplasm and endoplasm was observed. Strong surface attachment in the area between the middle and anterior of the cell a the glass slide was observed (Supplementary Video 6). The cell glided in the anterior direction.

**Fig. 3:**
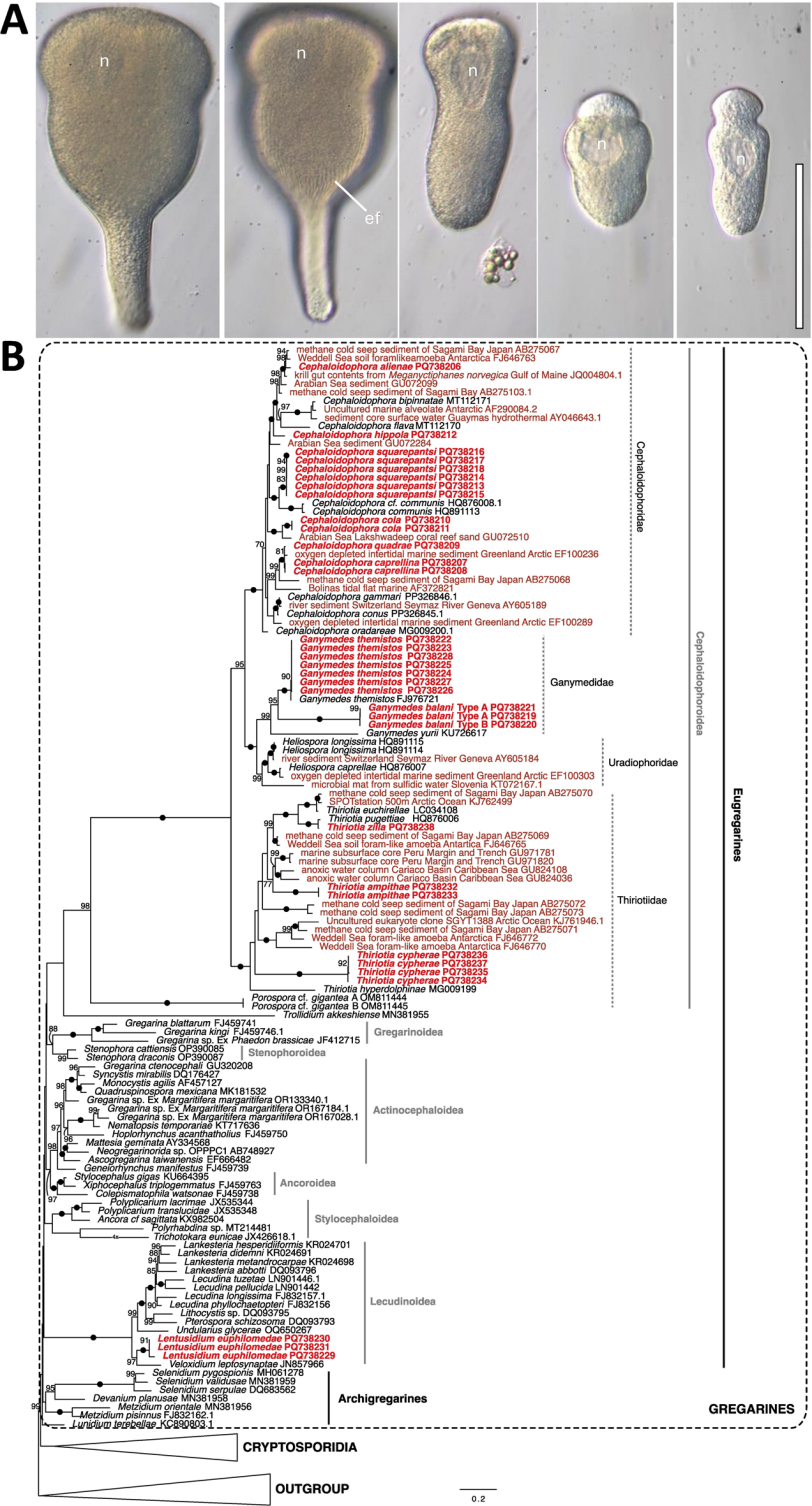
Light micrographs of *Lentusidium euphilomedae* n. gen. et sp. and maximum likelihood phylogenetic tree of Apicomplexa. (**A**) *Lentusidium euphilomedae* n. gen. et sp. trophozoites, nucleus (n), and epicytic folds (ep). The two images on the left are of the same individual cell taken at different planes of view through the cell. Scale bar = 100 µm. (**B**) Nuclear SSU + LSU rRNA gene phylogenetic tree representing major gregarine superfamilies generated using the GTR + F + R10 substitution model, 1000 UFBs, and 138 taxa. Bootstraps under 70% are omitted. Gregarines are highlighted with a dotted box. The tree is rooted on Dinoflagellates. A fully detailed tree is shown in Supplementary Fig. S2. Black circle = Nodes with full bootstrap support. Red bolded text = Newly added lineages. Brown text = Sequences from environmental or microbiome surveys.

*Lentusidium euphilomedae* n. gen. et sp. trophozoites were collected from the seed shrimp *Euphilomedes* sp. (Fig. 3A). Cells of various shapes were approximately 157.5 µm in length and 84 µm in width. Larger cells resembled a guitar where the anterior was widest, narrowing to a thin long posterior that was rounded at the end. The posterior slowly extended and retracted into the larger portion of the trophozoite. The cell surface had epicytic folds. Smaller cells (length < 50 µm) also had a wider anterior that narrowed in the middle and returned to the same width towards the posterior. The trophozoites exhibited slow metaboly-like ability to change its shape completely; morphing into a ball or acorn-shaped cell. These features differed significantly from other crustacean-infecting gregarines. The nucleus was between the anterior and centre region of the cell.

#### SSU + LSU rDNA phylogenetic analyses

A maximum-likelihood (ML) SSU-LSU rDNA phylogenetic tree was generated with 138 taxa consisting of gregarines representing superfamilies with molecular data available, environmental samples proposed to branch within the Cephaloidophoroidea, and BLASTn hits corresponding to new sequences added during this study (Fig. 3B, Supplementary Fig. S2). Despite a lack of clear sequence similarity, a number of environmental samples clustered with several of our new gregarines (Fig. 3B). Within the Cephaloidophoridae clade *Cephaloidophora alienae* n. sp. branched in a group composed of environmental samples consisting of sediment collected near Japan^41^, deep-sea foraminifera-like amoeba from Antarctica^42^, gut contents of krill from the Gulf of Maine^43^, and sediment from the Arabian sea^44^ (Fig. 3B). This was sister to a clade formed by *C. bipinnatae*, *C. flava*, environmental samples from Antarctica^45^, and hydrothermal vents^46^ with full bootstrap support (100%). *Cephaloidophora hippola* n. sp. emerged at the base of this clade with full bootstrap support (100%) (Fig. 3B). Sister to this collective group was a clade consisting of *Cephaloidophora squarepantsi* n. sp. branching sister to *C. communis* with full bootstrap support (100%) (Fig. 3B). *Cephaloidophora cola* n. sp. branched sister to coral reef sand collected from the Arabian Sea^44^, and collectively branched sister to the clade containing *C. alienae* n. sp.*, C. hippola* n. sp., and *C. squarepantsi* n. sp. and closely branching environmental samples. *Cephaloidophora caprellina* n. sp. branched with *C. quadrae* n. sp. and an intertidal marine sediment sample from Greenland^47^ which collectively branched sister to *C. gammari*, *C. conus*, river sediment from Switzerland^48^, and another sediment sample from Greenland^47^. *Cephaloidophora oradareae* branched sister to the other *Cephaloidophora* species and environmental samples collectively (Fig. 3B). *Ganymedes balani* n. sp. branched sister to *G. themistos* with strong support (95%), with *G. yurii* branching sister to the pair (Fig. 3B). *Thiriotia ampithae* n. sp. branched in a group with water column and sediment samples from Japan^41^, Antarctica^45^, the Peru Trench^49^, and the Caribbean Sea^50^. This clade, together with *T. ampithae* n. sp., branched sister to a group composed of *T. euchirellae, T. pugettiae*, *T. zilla* n. sp.*,* sediment samples from Japan^41^, and the Arctic Ocean^51^. *Thiriotia zilla* n. sp. branched sister to *T. pugettiae* with full bootstrap support (100%) (Fig. 3B). *Thiriotia cypherae* n. sp. emerged as sister to these *Thiriotia* species and environmental samples that branched within the clade. *Thiriotia hyperdolphinae* emerged as sister to this collective group (Fig. 3B). Unexpectedly, *Lentusidium euphilomedae* n. gen. et sp. and *Veloxidium leptosynaptae* formed a fully supported (100%) clade sister to all other Lecudinoidea. Cephaloidophoroidea branched sister to a clade formed by Gregarinoidea and Stenophoroidea with low bootstrap support (54%) (Fig. 3B). The branch positions of the novel species described this study was reflected in a broader ML phylogenetic tree generated using SSU-LSU rDNA gene sequences from 131 published apicomplexans and sequences extracted from 33 new transcriptomes. This tree included all known major apicomplexan taxonomic groups (Supplementary Fig. S3). In this phylogenetic tree, Cephaloidophoroidea branched sister to Lecudinoidea with moderate bootstrap support (79%) (Supplementary Fig. S3). Both phylogenetic trees showed Cephaloidophoridae branching sister to a clade formed by Ganymedidae and Uradiophoridae, with *Thiriotia* branching sister to the three clades, and *Porospora* branching sister to all members of the Cephaloidophoroidea collectively (Fig. 3B, Supplementary Fig. S3).

### Phylogenomic analyses

**Fig. 4:**
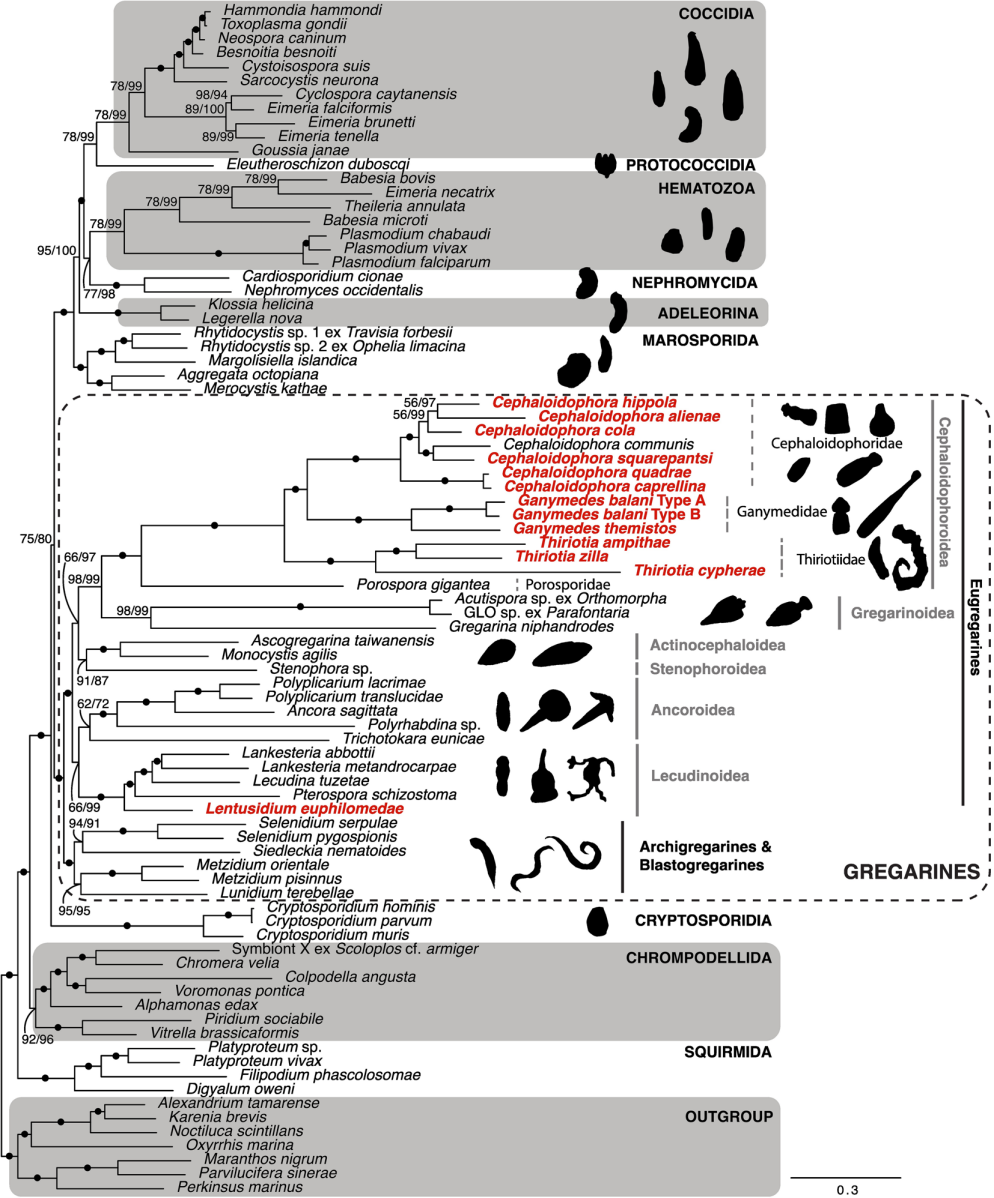
Maximum likelihood phylogenomic tree of Apicomplexa generated using 85 taxa and inferred from 190 nuclear proteins (45,976 amino acid sites) under the LG + C60 + F + G mixture model with 1000 UFBs. Alternating grey boxes and blank areas indicate major Apicomplexan subgroups. Gregarines are highlighted with a dotted box. The tree is rooted on Dinoflagellates. Red bolded text = Gregarines from this work. Black circles = Nodes with 100% ultrafast bootstrap support and 100% posterior mean site frequency (PMSF) bootstrap support from 200 non-parametric bootstrap replicates, otherwise values are indicated as follows: ML/PMSF. Silhouettes depict representative morphologies.

Unresolved relationships within and between apicomplexan groups are common in published phylogenetic analyses^34,52,53^. However, phylogenomic analyses have successfully clarified poorly supported nodes and apicomplexan evolution, more generally^12,54,55^. To this end, we generated a ML phylogenomic tree based on 85 apicomplexan taxa and 190 proteins to assess the position of the newly characterized gregarines relative to other apicomplexans. These analyses indicate that the lineage, Cephaloidophoridae, composed of *Cephaloidophora hippola* n. sp.*, C. alienae* n. sp.*, C. cola* n. sp.*, C. squarepantsi* n. sp., *C. quadrae* n. sp.*,* and *C. caprellina* n. sp. formed a fully-supported clade with *C. communis* (Fig. 4). The addition of new molecular data from Ganymedidae and Thiriotiidae, represented by *G*. *themistos* and *G. balani* n. sp.*,* and *T. ampithae* n. sp.*, T. zilla* n. sp.*,* and *T. cypherae* n. sp. (respectively)*,* confirm their relative position within the Cephaloidophoroidea. Our phylogenomic tree and SSU-LSU rDNA phylogenetic tree of gregarines show Gregarinoidea as the closest branching sister group to Cephaloidophoroidea (Figs. 3B, 4) while our SSU-LSU rDNA phylogenetic tree of apicomplexans show Lecudinoidea as the closest branching sister group (Supplementary Fig. S3). As in our two SSU-LSU rDNA phylogenetic trees, the multi-gene phylogenomic tree shows *Lentusidium euphilomedae* n. gen. et sp. does not branch with members of the Cephaloidophoroidea but instead with the Lecudinoidea, forming a sister lineage to all pre-existing isolates (*Pterospora schizostoma*, *Lankesteria abbottii*, *L. metandrocarpae*, and *Lecudina tuzetae)* with full bootstrap support (100%) (Fig. 4).

## Discussion

### Taxonomic history and remarks on morphology

*Cephaloidophora* was established with characters of the family, Cephaloidophoridae, where the trophozoites are septate, epimerite is small, syzygy is caudo-frontal, and stages are intracellular and extracellular^39^. Hosts are crustaceans including Cirripedia, Copepoda, Euphausiacea, Amphipoda, and Decapoda. Initially the genus appeared to be identical with *Frenzelina,* but this name was abandoned for already being in use leading to the full adoption of *Cephaloidophora*^39^. Several species of *Rotundula*, including those initially placed in the *Gregarina,* and *Pyxinoides chtalami* have been reassigned to *Cephaloidophora*, which overall contains over 70 described species^39^. Prior to this study, molecular data had only been obtained from 6 species. *Caridohabitans* is the only other genus in this family and is differentiated by a crescent-shaped epimerite, but no molecular data exists for any members and the justification of the genus itself has been questioned^39^. The novel *Cephaloidophora* species identified in this study were found in Amphipoda and Decapoda hosts that were not previously identified as hosts of *Cephaloidophora* gregarines. Consistent with the genus characters *Cephaloidophora hippola* n. sp. has a small epimerite and caudo-frontal syzygy like *C. oradareae* and *C. communis*^56^*. Cephaloidophora alienae* n. sp. and *C. quadrae* n. sp. were septate and exhibited caudo-frontal syzygy. *Cephaloidophora cola* n. sp. had an inconspicuous epimerite and discreet septa similar to *C. bipinnatae*^38^. *Cephaloidophora squarepantsi* n. sp. cells had a blunted cell anterior and posterior causing a square-like appearance. Blunted cell posteriors also appear in *C. flava* and *C. communis*^38^. The satellites of *C. quadrae* n. sp. and *C. caprellina* n. sp. were smaller than the primite like in *C. flava*^38^. *Cephaloidophora alienae* n. sp. and *C. caprellina* n. sp. had rounded globular anteriors and posteriors similar to the morphology of *C. bipinnatae*, *C. pacifica*, and *C. gammari*, contrasting *Thiriotia* and *Ganymedes* cells that are commonly elongated^38,57^. Jerky or pulsing movements observed in *C. alienae* n. sp. and *C. squarepantsi* n. sp. (Supplementary Table S2) have also been observed in *C.* cf. *communis* which exhibits various modes of motility differentiating it from typical apicomplexan gliding machinery where with the architecture of epicytic folds, internal lamina, and a mucopolysaccharide coating contribute to motility^58,59^. *Thiriotia* was erected with characters of the family, Thiriotiidae, where trophozoites are aseptate with a rounded anterior, syzygy is caudo-frontal or fronto-lateral^39^. It is the sole genus of the family. Hosts include crustaceans belonging to the Decapoda, Copepoda and Amphipoda. The type species, *Thiriotia pisae*, was transferred from the genus *Porospora* due to the aseptate morphology of trophozoites^39^. Among the four reported species in this genus molecular data has been obtained from three: *T. pugettiae*. *T. euchirellae*, and *T. hyperdolphinae*. The three novel *Thiriotia* species identified in this study were found in newly identified Decapoda and Amphipoda hosts. The epimerite of *T. ampithae* n. sp. and *T. cypherae* n. sp. trophozoites appeared decorated like *T. euchirellae* and smooth in others like *T. zilla* n. sp., *T. pisae*, and *T. pugettae*^21,39,60^. *Thiriotia cypherae* n. sp. was elongated, had various morphologies, exhibited creases along the cell, had caudo-frontal syzygy, and possessed either a rounded or blunted epimerite similar to variations observed in *T. pisae*^39^. However, *T. cypherae* n. sp. trophozoites were shorter and had rounded and flattened cell posteriors. *Thiriotia zilla* n. sp. trophozoites were elongated with enlarged rounded epimerites, had a shallow indentation below the epimerite, and a narrowed cell posterior similar to that observed in *T. pugettiae* and *T. pisae*^39^. In contrast to other members of the genus, *T. zilla* n. sp. was larger, narrower on one side, and had a lobulated surface texture. Gigantic gregarines in the superfamily Cephaloidophoroidea have been reported in the form of *P. gigantia* which measures up to 16 mm^61^ and *T. pugettiae* which measures up to 2 mm in length^21^. The size of *T. zilla* n. sp. was similarly extraordinary, measuring 2 mm (Supplementary Table S2) and reaching the previous recorded maximum for *Thiriotia.* At the other end of extreme sizes, *T. ampithae* n. sp. measured as small as 50 µm, barely reaching the diameter of some *T. zilla* n. sp. nuclei by comparison (Supplementary Table S2). *Thiriotia zilla* n. sp. moved quickly with gliding speeds similar to the fast speeds observed in *Porospora gigantia*^21^. Further, the gliding motility of *T. zilla* n. sp. appeared like it was floating through liquid rather than attached to a substrate or perhaps attached at one part of the cell as it was able to bend and curl at the same time as moving in one direction (Supplementary Video 3, 4). Curling and twisting motions exhibited by *T. zilla* n. sp. was also reported in *T. pugettiae* which curled into spirals and balls^21^. *Ganymedes* is the sole genus of the family Ganymedidae, where trophozoites are aseptate, elongate, and cylindrical, the anterior epimerite is globular or sucker-like, and syzygy is caudo-frontal or lateral^39^. Hosts are freshwater and marine members of the Cirripedia, Copepoda, Syncardia, Mysidacea, Amphipoda, and Decapoda. Although the family is characterized as aseptate, the genus is regarded as an intermediate between septate and aseptate gregarines due to the epimerite of the type species, *Ganymedes anaspidis*, which exhibits a ball-like bulge in the epimerite resulting in a septum-like indentation^39^. In *G. haeckeli* a transitory septum is observed in trophic life stages^39^. A number of gregarines from several genera including *Paraophioidina, Monocystis*, *Uradiophora*, have been reassigned to this genus^39^. *Ganymedes balani* n. sp. was cylindrical, had caudo-frontal syzygy like *G. yurii* and *G. anaspidis*, and was found in a Cirripedia host^22,39^. The gamonts of *G. balani* n. sp. resembled early *G. haeckeli* trophic stages which had a transitory septum^39^. The epimerite of *G. balani* n. sp. formed internal lobe-like structures in the ectoplasm similar to illustrations of *Pyxinoides bolitoides* and *Gregarina valettei*^62^, both also isolated from barnacles, but ultimately differed by size, morphology, and host species. At the syzygy junction the overlap between the gamonts resembled the ball-in-cup-like syzygy reported in *G. anaspidis* and *G. themistos*^39,40^. Gliding observed in *G. balani* n. sp. has also been reported in *G. yurii*^22^. *Lentusidium euphilomedae* n. gen. et sp. morphology differed from members of the Cephaloidophoroidea most prominently by demonstrating dynamic changes in cell shape. Bending has been reported in *C. communis*^58^ and was observed in *C. quadrae* n. sp., *T. cypherae* n. sp., and *T. zilla* n. sp., but metaboly-like activity is not characterized in members of the Cephaloidophoroidea^39^. The metaboly-like activity observed in *L. euphilomedae* n. gen. et sp. was similar to but slower than the strong peristaltic cytoplasm movement in species belonging to the Lecudinoidea: *Pterospora schizostoma, P. floridiensis,* and *Urospora ovalis*^63,64^. The Lecudinoidea is a superfamily of gregarine apicomplexans with diverse hosts including annelids, nemertians, echinoderms, and gastropods^13,34^. The trophozoites of species belonging to the Lecudinoidea are aseptate and exhibit a wide range of behaviours including gliding, bending, twisting, and peristaltic movements^13,34^. Some *L. euphilomedae* n. gen. et sp. resembled Cephaloidophoroidea morphology with where an anterior bulge created a septum-like indentation, but this may have formed transiently to anchor the gregarine to the host. Comparisons of ultrastructural features are needed to determine they are homologous or analogous, and to compare eugregarine epimerites in general, which are especially understudied in the Cephaloidophoroidea^34^.

The septum is a cellular feature of gregarines that divide the trophozoite cell into distinct regions and was historically used to classify families within the Cephaloidophoroidea as either aseptate or septate^39^. Historically aseptate gregarines were not considered to possess a septum. Septate gregarines possess either one septum that divides the protomerite and the deutomerite or two septa dividing the epimerite, protomerite, and deutomerite. For aseptate gregarines the attachment organelle at the anterior of the cell was once referred to as a “mucron” for both archigregarines and aseptate eugregarines, however ultrastructural findings have resulted in a proposal to restrict the term “mucron” to archigregarines and refer to this structure in aseptate eugregarines as the “epimerite”^65^. Additional morphological observations of gregarine apicomplexans, as well as phylogenetic analyses, have revealed that there may be exceptions to the historical use of septa as a classification character in Ganymedidae (aseptate), Thiriotiidae (aseptate), Cephaloidophoridae (septate), and Uradiophoridae (septate)^39^. A septum has been reported in specific life stages in Ganymedidae species^39^. Septum-like indentations were observed in Thiriotiidae species^39^. Discreet transitory septa have been reported in Cephaloidophoridae and Uradiophoridae demonstrating that this feature may be more dynamic in this group than previously thought^39^.

Taxonomic definitions of crustacean-infecting apicomplexans vary in detail. Morphological similarities and the discovery of transient morphologies present in specific life cycle stages thought to be characteristic of other taxonomic groups have confounded morphological classifications in this group^39^. Several crustacean-infecting gregarines have been transferred to the Cephaloidophoroidea, and others have been transferred between genera in the superfamily over the last century^39^. Most crustacean-infecting gregarines lack associated molecular data to confirm classifications. When available, molecular data has helped to resolve evolutionary relationships, as we have in this study. Taxonomic definitions remain vague and non-specific for a variety of factors including limited access to hosts, few culturing methods for both hosts and gregarines alike, and a greater diversity of observed morphological characters relative to initial taxonomic descriptions. Furthermore, patterns of shared traits are unlikley to appear when only a small fraction of organisms are identified^2,26^. There are likely several unstudied gregarines yet to be collected, identified, and characterized, especially at the molecular level, and those from marine environments and hosts are especially understudied despite representing the majority of apicomplexan diversity according to environmental surveys. Unidentified species detected in environmental surveys that branch within the Cephaloiophoroidea span across freshwater to marine environments, and geographically from the Atlantic to the Antarctic. The introduction of molecular phylogenetics has greatly assisted in confirming classifications, species identification, and providing evidence of evolutionary relationships and is expected to continue significantly improving the accuracy of classifications and our understanding of gregarine evolutionary relationships^21,38,39,56^. Support for the 11 novel species we report is justified by a combination of morphological observations and host identity and further substantiated by compelling molecular data. Specifically, species identification was assessed by comparing isolated specimens to published morphological descriptions of known gregarines, distinct host identity (due to the host specificity of gregarines), and further clarified by comparing rRNA sequences (both SSU and LSU rRNA) extracted from assembled transcriptomes (using a 99% identity threshold to designate species identity^30^) and phylogenetic proximity. The highest percentage identity shared between the majority of new species identified in this study and gregarines in the NCBI GenBank nr database, and notably species within the same genus, was 80–90% suggesting high divergence from described species already deposited the database which are represented on our phylogenetic and phylogenomic trees.

### Crustacean-associated apicomplexans are not restricted to the Cephaloidophoroidea

These new transcriptomic datasets expand the representation of the superfamily Cephaloidophoroidea in phylogenomic analyses. Previously, only four taxa represented the Cephaloidophoroidea: *Cephaloidophora communis, Cephaloidophora* sp*., Heliospora caprellae,* and *Porospora gigantea*. We have now increased this to 15. Although these data generally conform to the idea that crustacean-associated apicomplexans likely belong to the Cephaloidophoroidea, here we demonstrate that this is not an absolute rule. *Lentusidium euphilomedae* n. gen. et sp. represents a new level of diversity of crustacean-infecting gregarines, branching as sister to *Veloxidium leptosynaptae* within the Lecudinoidea. More genomic data from deep-branching gregarines could clarify whether there is a larger group of crustacean-infecting gregarines in the lecudinoideans, or if this was instead a recent case of host jumping.

## Conclusions

Here, several novel gregarines found in a variety of crustacean hosts expand the known diversity of Cephaloidophoroidea, Lecudinoidea, and crustacean-associated apicomplexans. Cephaloidophoroidea are highly abundant in marine ecosystems^2^ but underrepresented in phylogenomic analyses. Exploration of crustacean-associated apicomplexans has been limited compared to other apicomplexan lineages. Our work contributes to establishing a more balanced representation of apicomplexan diversity to interpret apicomplexan evolution which, historically, has been biased towards terrestrial (particularly clinical) lineages, creating a highly uneven representation of diversity. Future work on Cephaloidophoroidea and crustacean gregarines beyond cell biology, diversity, evolution could explore its ecological role, host interactions, marine food web dynamics, and impact on the fishing industry, and will elucidate the significance of these ubiquitous yet enigmatic apicomplexans. The description of a new isolate branching outside of the Cephaloidophoroidea superfamily, indicates unsampled diversity and the potential for host switching. These data demonstrate the importance of studying apicomplexan lineages beyond commonly known clinically relevant isolates, showing crustacean gregarines do not all phylogenetically group within the Cephaloidophoroidea, as previously thought.

## Taxonomic summary

Superphylum Alveolata Cavelier-Smith, 1991

Phylum Apicomplexa Levine, 1970

Class Gregarinea Bütschli 1882, stat. nov. Grassé 1953

Order Eugregarinorida Léger, 1900

Superfamily Cephaloidophoroidea Kamm, 1922

Family Cephaloidophoridae Kamm, 1922

Genus *Cephaloidophora* Mavrodiadi, 1908

### *Cephaloidophora hippola* n. sp. Na and Keeling

**Diagnosis.** Gamonts are capsule-shaped, 33.5 µm long, and 22 µm wide with rounded edges and flattened sides. Gamonts pair in caudo-frontal syzygy. Primite and satellite are divided into the protomerite and deutomerite by a discreet septum. A septum separates the epimerite and protomerite in the primite. The nucleus is 11.5 µm in diameter centrally located. The cell is colourless. Large granular material is in the cytoplasm. A clear separation demarks the endoplasm and ectoplasm. Cell motility not observed.

**Gene sequence.** A sequence of the rRNA is deposited in GenBank: PQ738212. A raw transcriptome sequence is deposited in NCBI SRA: BioProject PRJNA1195655.

**Locality.** 50°06′56.6"N 125°13′16.4"W, Hyacinthe Bay, Quadra Island, British Columbia, Canada.

**Type habitat.** Marine.

**Host.** *Hippolyte* sp. Leach 1814 (Metazoa, Arthropoda, Crustacea, Decapoda, Hippolytidae).

**Location in host.** Intestine.

**Holotype.** Figure 1A. Physical specimen annihilated during preparation.

**Zoobank Registration LSID.** urn:lsid:zoobank.org:act:324BB859-2FE5-4196-ADB6-81F9AE7C8CE9.

**Etymology.** The species name *hippola* refers to the host genus.

### *Cephaloidophora caprellina* n. sp. Na and Keeling

**Diagnosis.** Trophozoite and gamonts are capsule-shaped with a rounded anterior and posterior. The primite gamont and trophozoite are approximately 69 µm in length and 42 µm in width. Gamonts pair in caudo-frontal syzygy. A septum separates the protomerite and deutomerite. The deutomerite is darkly pigmented, obscuring the position of the nucleus. The satellite is smaller than the primite measuring 52 µm in length and 27 µm in width. A thick colourless ectoplasm surrounded the darkly pigmented endoplasm of the primite gamont and trophozoite. Cell motility not observed.

**Gene sequence.** Sequences of the rRNA are deposited in GenBank: PQ738207 and PQ738208. Raw transcriptome sequences are deposited in NCBI SRA: BioProject PRJNA1195655.

**Locality.** 50°06′10.5"N 125°12′41.1"W, Heriot Bay, Quadra Island, British Columbia, Canada.

**Type habitat.** Marine.

**Host.** *Caprella* sp. Lamarck 1801 (Metazoa, Arthropoda, Crustacea, Amphipoda, Caprellidae).

**Location in host.** Intestine.

**Holotype.** Figure 1B,C. Physical specimen annihilated during preparation.

**Zoobank Registration LSID.** urn:lsid:zoobank.org:act:F759891A-7417–4439-B2EE-F1110EF2F3AF.

**Etymology.** The species name *caprellina* refers to the host genus.

### *Cephaloidophora alienae* n. sp. Na and Keeling

**Diagnosis.** Gamonts are spherical with an anterior that extends to a pointed end in the primite and flattened anterior with sharp edges in the satellite. Gamont measures approximately 41 µm in length and 39.5 µm in width. Gamonts pair in caudo-frontal syzygy. Nucleus is 13 µm diameters, round, and centrally located in the satellite. A septum separates the protomerite and deutomerite. In the satellite the protomerite is less pigmented than the deutomerite. A division between the endoplasm and ectoplasm can be seen. The gregarine moves in the anterior direction with pulsing movements.

**Gene sequence.** A sequence of the rRNA is deposited in GenBank: PQ738206. A raw transcriptome sequence is deposited in NCBI SRA: BioProject PRJNA1195655.

**Locality.** 50°06′58.4"N 125°13′16.8"W, Hyacinthe Bay, Quadra Island, British Columbia, Canada.

**Type habitat.** Marine.

**Host.** *Alienacanthomysis macropsis* Tattersall 1932 (Metazoa, Arthropoda, Crustacea, Mysidacea, Mysidae).

**Location in host.** Intestine.

**Holotype.** Figure 1D. Physical specimen annihilated during preparation.

**Zoobank Registration LSID.** urn:lsid:zoobank.org:act:689EE2D5-1E28-44DE-B31D-777E401A51C8.

**Etymology.** The species name *alienae* refers to the host genus.

### *Cephaloidophora quadrae* n. sp. Na and Keeling

**Diagnosis.** Gamonts are cylindrical with a small domed anterior and rounded posterior. Gamonts pair in caudo-frontal syzygy where the primite and satellite overlap. Satellite is smaller than the primite. Primite is 15.5 µm long and 7.5 µm wide. Satellite is 11 µm long and 5 µm wide. Discreet septum separates the protomerite and deutomerite. Primite lightly pigmented, satellite colourless. The spherical 6 µm diameter nucleus of the primite is in the cell posterior directly above the syzygy junction. Primite has an ectoplasm and endoplasm. Gliding motility observed as well as repetitive bending movements in the primite.

**Gene sequence.** A sequence of the rRNA is deposited in GenBank: PQ738209. A raw transcriptome sequence is deposited in NCBI SRA under BioProject PRJNA1195655.

**Locality.** 50°06′58.1"N 125°13′17.1"W, Hyacinthe Bay, Quadra Island, British Columbia, Canada.

**Type habitat.** Marine.

**Host.** A species of *Caprella* Lamarck 1801 (Metazoa, Arthropoda, Crustacea, Amphipoda, Caprellidae).

**Location in host.** Intestine.

**Holotype.** Figure 1E. Physical specimen annihilated during preparation.

**Zoobank Registration LSID.** urn:lsid:zoobank.org:act:146C88A8-B995-4EB8-81E0-2B6C0AE3121D.

**Etymology.** The species name *quadrae* refers to the name of the island where the host was sampled.

### *Cephaloidophora cola* n. sp. Na and Keeling

**Diagnosis.** Trophozoites are elongated and cylindrical. Length 101 µm and width 23.5 µm, narrowing to 35 µm at the anterior, and 38 µm at the posterior. Anterior rounded with discreet septum. The 11 µm diameter nucleus is round and centrally located. Pigmentation is observed in the middle of the cell and increases in intensity towards the posterior. Partitioning of the endoplasm and ectoplasm. Cell anterior undulates, no other motility observed.

**Gene sequence.** Sequences of the rRNA are deposited in GenBank: PQ738210 and PQ738211. A raw transcriptome sequence is deposited in NCBI SRA: BioProject PRJNA1195655.

**Locality.** 50°06′10.5"N 125°12′41.1"W, Heriot Bay, Quadra Island, British Columbia, Canada.

**Type habitat.** Marine.

**Type host.** *Eriocheir sinensis* H. Milne Edwards 1853 (Metazoa, Arthropoda, Crustacea, Decapoda, Varunidae).

**Location in host.** Intestine.

**Holotype.** Figure 1F. Physical specimen annihilated during preparation.

**Zoobank Registration LSID.** urn:lsid:zoobank.org:act:77E2A136-7E61-462D-B315-F7B1341ABEAD.

**Etymology.** The species is named *cola* for the resemblance of trophozoites to 1950s glass cola bottles.

### *Cephaloidophora squarepantsi* n. sp. Na, Jacko-Reynolds, and Keeling

**Diagnosis.** Trophozoites are rectangular-prism shaped with flat sides and rounded corners. Cells 89.3 µm long and 81.3 µm wide. The narrower side of the cell measures 20 µm wide. Some cells observed with shallow indents on sides, rounded anterior, a bulge protruding from the cell centre. A septum separates the protomerite from the deutomerite. Nucleus is 23.3 µm in diameter and located in the cell posterior. Pigmentation varies between cells. Cells moved by pulsing motility in the anterior direction.

**Gene sequence.** Sequences of the rRNA are deposited in GenBank: PQ738213- PQ738218. Raw transcriptome sequences are deposited in NCBI SRA: BioProject PRJNA1195655.

**Locality.** 50°06′57.8"N 125°13′17.3"W, Hyacinthe Bay, Quadra Island, British Columbia, Canada.

**Type habitat.** Marine.

**Type host.** A species of *Neomysis* sp. Czerniavsky 1882 (Metazoa, Arthropoda, Crustacea, Malacostraca, Mysidae).

**Location in host.** Intestine.

**Holotype.** Figure 1H–J. Physical specimen annihilated during preparation.

**Zoobank Registration LSID.** urn:lsid:zoobank.org:act:7D8DE2DC-55D8-4593-863A-47722FB0A7AC.

**Etymology.** The species is named after the main rectangular-prism shaped character, Spongebob Squarepants, of a popular children’s animated television series created by a former marine science educator due to the resemblance in shape between the trophozoite and the fictional character.

Family Thiriotiidae Desportes and Schrével 2013

Genus *Thiriotia* Desportes, Vivarès and Théodoridès 1977

### *Thiriotia ampithae* n. sp. Na and Keeling

**Diagnosis.** Trophozoites are long and cylindrical with a rounded posterior. The trophozoites measure 53.5 µm long and 10 µm wide. Trophozoites are decorated with crown-like features or a small, rounded protruding epimerite separated at an indentation from the remainder of the cell. A 5.5 µm diameter nucleus is situated halfway between the anterior and centre of the cell. Cell motility not observed.

**Gene sequence.** Sequences of the rRNA are deposited in GenBank: PQ738232 and PQ738233. Raw transcriptome sequences are deposited in NCBI SRA: BioProject PRJNA1195655.

**Locality.** 50°06′58.4"N 125°13′16.8"W, Hyacinthe Bay, Quadra Island, British Columbia, Canada.

**Type habitat.** Marine.

**Type host.** *Ampithoe valida* S. I. Smith 1873 (Metazoa, Arthropoda, Crustacea, Amphipoda, Amphithoidae).

**Location in host.** Intestine.

**Holotype.** Figure 2A,B. Physical specimen annihilated during preparation.

**Zoobank Registration LSID.** urn:lsid:zoobank.org:act:5087CC43-944D-467E-AE14-34CFCA75D5B5.

**Etymology.** The species name *ampithae* refers to the host genus.

### *Thiriotia cypherae* n. sp. Na, Jacko-Reynolds, Holt, and Keeling

**Diagnosis.** Trophozoites and gamonts long, cylindrical, and vary in morphology. Anterior and posterior flattened with rounded edges. Syzygy caudo-frontal. Trophozoites 227 µm long and 37.5 µm wide. Cell surface creased in various locations throughout some cells, others are smooth. Trophozoite may exhibit short protrusions at the anterior or posterior. Trophozoite nucleus is round, 21.5 µm in diameter, and centrally located. Gamonts are approximately 242 µm in length and 38.5 µm in width. Primite anterior colorless. Cell pigmentation darkens towards the posterior. Gamont nucleus is 26 µm in diameter and centrally located. Ectoplasm and endoplasm partitioned. Gliding motility in the anterior direction.

**Gene sequence.** Sequences of the rRNA are deposited in GenBank: PQ738234- PQ738237. Raw transcriptome sequences are deposited in NCBI SRA: BioProject PRJNA1195655.

**Locality.** 50°06′57.8"N 125°13′17.3"W, Hyacinthe Bay, Quadra Island, British Columbia, Canada.

**Type habitat.** Marine.

**Type host.** *Cyphocaris challenger* Stebbing 1888 (Metazoa, Arthropoda, Crustacea, Amphipoda, Cyphocarididae).

**Location in host.** Intestine.

**Holotype.** Figure 2C–E. Physical specimen annihilated during preparation.

**Zoobank Registration LSID.** urn:lsid:zoobank.org:act:72A7869A-4DCB-4F0B-9966-2063379F4EAF.

**Etymology.** The species name *cypherae* refers to the host genus.

### *Thiriotia zilla* n. sp. Na and Keeling

**Diagnosis.** Trophozoites vermiform, 1877 µm long, and 154 µm wide. Cells taper at the anterior and posterior and have a narrower side measuring 49 µm in width. Nucleus is 47.6 µm in diameter and located in the anterior of the cell. Lobulated surface texture resembling wrinkles appear throughout the cell. Cells can bend, twist, and curl and are overall flexible. Gliding motility is fast and in the anterior direction.

**Gene sequence.** A sequence of the rRNA is deposited in GenBank: PQ738238. A raw transcriptome sequence is deposited in NCBI SRA: BioProject PRJNA1195655.

**Locality.** 48°24′10.0"N 123°20′54.6"W, Clover Point, Victoria, British Columbia, Canada.

**Type habitat.** Marine.

**Type host.** A species of *Scyra* Dana 1851 (Metazoa, Arthropoda, Crustacea, Decapoda, Epialtidae).

**Location in host.** Intestine.

**Holotype.** Figure 2F–H. Physical specimen annihilated during preparation.

**Zoobank Registration LSID.** urn:lsid:zoobank.org:act:A3FD438A-2D89-4802-85D4-06483897CFBE.

**Etymology.** The species name *zilla* is derived from the fictional monster, Godzilla, given the staggering size and wrinkly textured surface of the trophozoites.

Family Ganymedidae Huxley 1910

Genus *Ganymedes* Huxley 1910

### *Ganymedes balani* n. sp. Na and Keeling

**Diagnosis.** Gamonts are joined in caudo-frontal syzygy. Type A cells are approximately 65.5 µm long and 26 µm wide. Type B cells are approximately 51.3 µm long and 20.3 µm wide. The epimerite narrows to a onion-dome shaped anterior. A septum-like indentation below the epimerite is 4 µm narrower than the cylindrical body of the cell which has a rounded posterior. A rounded nucleus (diameter = 12 µm for Type A; 9 µm for Type B) sits in the centre-to-posterior area of the gamonts. Some gamont pairs overlap at the anterior of the satellite and posterior of the primite. A clear separation appears between the ectoplasm and endoplasm. Cells are mostly colourless, with small granules in the endoplasm. Movement occurs via gliding motility in the anterior direction.

**Gene sequence** Sequences of the rRNA are deposited in GenBank: PQ738219- PQ738221. Raw transcriptome sequences are deposited in NCBI SRA: BioProject PRJNA1195655.

**Locality.** 50°06′56.6"N 125°13′16.4"W, Hyacinthe Bay, Quadra Island, British Columbia, Canada.

**Type habitat.** Marine

**Type host.** *Balanus glandula Darwin, 1854* (Metazoa, Arthropoda, Crustacea, Balanomorpha, Balanidae).

**Location in host.** Intestine.

**Holotype.** Figure 2I,J. Physical specimen annihilated during preparation.

**Zoobank Registration LSID.** urn:lsid:zoobank.org:act:0F6E5514-A055-47E7-89A6-C3BFFBD5631B.

**Etymology.** The species name *balani* refers to the host genus.

Superfamily Lecudinoidea Cavalier-Smith, 2014, nom. nov. Simdyanov, 2017

Family Lecudinidae Kamm, 1922

### Genus *Lentusidium* n. gen. Na and Keeling

**Diagnosis.** Trophozoites exhibit slow metaboly allowing various cell shapes to take form; epicytic folds on surface; movement by gliding motility.

**Type species**. *Lentusidium euphilomedae* n. gen. et sp.

**Etymology.** The genus name *Lentusidium* refers to the slow movement by which the cells change shape as well as move.

### *Lentusidium euphilomedae* n. gen. et sp. Na and Keeling

**Diagnosis.** Trophozoites slowly morph into various shapes and sizes through metaboly. Cells are about 157.5 µm long and 84 µm wide. Larger cells (length > 100 µm) can resemble guitars with a thin protrusion from the cell resembling the neck that can extend and retract. Epicytic folds are on the surface of large trophozoites. Smaller sized cells (length < 100 µm) are vase, slipper, or acorn shaped with an indented region between the anterior and centre of the cell. Some cells are spherical. Nucleus anteriorly located in larger cells, between the anterior and centre in smaller cells, and can be round or oval-shaped measuring approximately 35.4 µm in diameter. Endoplasm and ectoplasm separated. Trophozoites move by gliding.

**Gene sequence** Sequences of the rRNA are deposited in GenBank: PQ738229- PQ738231. Raw transcriptome sequences are deposited in NCBI SRA: BioProject PRJNA1195655.

**Locality.** 50°06′54.4"N 125°13′13.9"W, Hyacinthe Bay, Quadra Island, British Columbia, Canada.

**Type habitat.** Marine.

**Type host.** A species of *Euphilomedes* sp. Poulsen 1962 (Metazoa, Arthropoda, Crustacea, Ostracoda, Philomedidae).

**Location in host.** Intestine.

**Holotype.** Figure 3A. Physical specimen annihilated during preparation.

**Zoobank Registration LSID**. urn:lsid:zoobank.org:act:9EE3AA54-4A0F-4DFA-BE6C-3168E0460599.

**Etymology.** The species name *euphilomedae* refers to the host genus.

## Materials and methods

### Sample collection and processing

Crustacean hosts were collected by sampling beaches, using deep sea plankton tows, and using hand-made benthic light traps set shoreside in localities along the South coast of British Columbia, Canada. Deep sea plankton tows were collected at 240 m depth. Benthic light traps were set overnight in the intertidal zones where complete coverage by water was maintained throughout deployment.

*Lentusidium euphilomedae* n. gen. et sp. was obtained from the seed shrimp *Euphilomedes* sp. found in light traps set at the Hakai Research Institute on Quadra Island in September 2020. *Lentusidium euphilomedae* n. gen. et sp. were also found in *Euphilomedes* sp. in light traps set in 2021 and 2022 but were not processed beyond obtaining and comparing SSU rRNA with 2020 samples for species identification. *Cephaloidophora caprellina* n. sp. was isolated from the skeleton shrimp *Caprella* sp., and *C. cola* n. sp. was obtained from juvenile forms of the Chinese mitten crab *Eriocheir* sp. collected from light traps set at Heriot Bay on Quadra Island in August 2021. *Cephaloidophora quadrae* n. sp. was obtained from skeleton shrimp *Caprella* sp., *C. hippola* n. sp. was found in a type of small shallow-water inhabiting shrimp *Hippolyte* sp., *C. alienae* n. sp. was obtained from a type of mysid, also known as an opossum shrimp, *Alienacanthomysis macropsis*, and *Thiriotia ampithae* n. sp. was isolated from the amphipod *Ampithoe valida* from light traps set at Hakai Research institute on Quadra Island in July, September, and August 2021, respectively. *Ganymedes balani* n. sp. was found in the acorn barnacle *Balanus glandula* from rocks picked up at a beach adjacent to the Hakai Research Institute on Quadra Island in September 2021. *Cephaloidophora squarepantsi* n. sp. was isolated from the mysid, also known as an opossum shrimp, *Neomysis* sp. in light traps in Victoria as well as Quadra Island in September 2021. *Thiriotia cypherae* n. sp. was found in the amphipod *Cyphocaris challenger* caught by deep sea plankton tows at Quadra Island in November 2021. *Thiriotia zilla* n. sp. was obtained from juvenile forms of the sharp-nosed crab *Scyra* sp. found in seagrass and mud found along the coast of Clover Point in Victoria in February 2022. *Ganymedes themistos* were found in the amphipod *Themisto libellula* caught in light traps set at Galiano Island in February 2022 (Supplementary Table S1).

Deep sea tow catches, light trap samples, and samples collected at the beach were stored at 4 ºC with bubblers until processing. Samples were processed within two days after collection (if not immediately). Marine crustaceans were stored in filtered seawater at 4 ºC until dissection. Prior to dissection, crustaceans were washed in the filtered seawater and then dissected in fresh filtered seawater. Amphipoda, Mysidacea, and soft-shelled Decapoda hosts were dissected using fine point tweezers to separate internal components. Decapoda, Balanomorpha, and Decapoda with hard shells were bisected using razors, followed by dissection and maceration using fine point forceps. The seed shrimp *Euphilomedes* sp. was released from its shell by applying pressure with blunt serrated forceps to break it apart prior to disssection and maceration using fine point foreceps. Single cells were imaged using a Leica DM IL LED inverted microscope and micrographs were taken with a Sony alpha 7RIII digital camera fixed to the microscope.

### Single cell isolation, transcriptome extraction, sequencing, and assembly

Hand-drawn glass micropipettes were used to isolate single cells resembling gregarines (visible nucleus, pigmented cytoplasm, cells in syzygy, cells exhibiting gliding or motility not observed in other surrounding cells). Cells were washed in filtered seawater up to two times before placing in 0.2 µl lysis buffer (Triton-X, RNaseOUT: USA) made according to instructions described in Picelli et al.^66^ and processed according to the proceeding Smart-Seq2^66^ protocol to obtain clean cDNA. The PCR amplification step of this process was adjusted to 23 cycles in order to account for low quantity starting cell material. Concentration of cDNA was quantified fluorometrically using Qubit and samples above a threshold of 0.2 ng/µl were used to make Nextera XT libraries using the DNA Library Preparation Kit (Illumina Inc., USA). All libraries were sequenced at the University of British Columbia Sequencing Centre on the Illumina NextSeq platform using 150 bp paired-end reads. Sequencing runs were repeated for 7 low-yield samples.

Raw reads were processed using FastQC v0.11.9 to assess read quality, TrimGalore v0.6.6 to remove adaptor sequences and low-quality bases, followed by SPAdes v3.15.1 to assemble transcriptome reads using the -rna option and default K-mer selected by SPAdes. To find the identity of each sample, nuclear SSU/LSU rRNA sequences were extracted using Barrnap v0.9 and queried using BLASTn against the NCBI GenBank nr database. Transcriptome samples that were the same species based on host type and BLASTn output (< 1% difference in Percent Identity) were reassembled together with SPAdes. An exception was *Ganymedes balani* n. sp. Type A and *Ganymedes balani* n. sp. Type B which were processed separately due to notable morphological differences between the two types for each species. Transcriptome completeness was estimated using BUSCO and the alveolate database to exclude samples with low coverage (< 8%)^67^. Contigs belonging to individual phyla were determined for each sample by running assembled reads through a megaBLAST search against the NCBI nr nucleotide database, then a diamond BLASTX search against the UniProt reference proteome database^68^. Phyla-based read dispersal was quantified then visualized using BlobTools and individual samples where contaminants exceeding apicomplexan reads by 75% were excluded from further analysis^69^. Open reading frames (ORFs) were predicted using TransDecoder v5.1.0 and longest ORFs were searched against the UniProt database using BLASTP to generate similarity-based annotations.

### Phylogenetic analysis

Nuclear SSU/LSU rRNA genes extracted from each sample for sample identification, as previously mentioned, and published sequences of apicomplexans identified from hosts and environmental samples retrieved from NCBI Genbank were aligned using MAFFT v7.481^70^. Then 5’ and 3’ ends were trimmed before using the trimA1 v1.4 with the -gt option for gap threshold set at 60% for the Apicomplexa dataset and 70% for the gregarine dataset^71^. To generate a maximum likelihood tree the substitution model GTR + F + R10, chosen by ModelFinder^72^, was used to build a maximum-likelihood tree using IQ-TREE v1.6.12 with 1000 ultrafast bootstrap replicates (UFB)^73^. The first tree was generated using 138 taxa representing gregarines, environmental samples, and microbiome sequences with Chrompodellids and Dinoflagellates as the outgroup. The second tree was generated using 164 taxa with representatives from all known major subgroups of apicomplexans and an outgroup comprised of Chrompodellids, Dinoflagellates, and Stramenopiles.

### Phylogenomic analysis

A set of 263 genes containing representatives from eukaryotic supergroups used in previous studies of apicomplexan phylogenomic analyses was used as a base upon which new transcriptomic data generated from this study was applied. Each of the 263 genes were used as a query to search using BLASTP against the new transcriptome coding sequences predicted by TransDecoder with an e-value threshold of 1 e-20 and query coverage threshold of 50%. Outputs were queried against the UniProt database using BLASTP to identify and remove poorly aligned sequences. Each gene in the finalized 263 gene set, which included the new transcriptome data, was then aligned using MAFFT L-INS-i^72^, trimmed with -gt 0.8 in trimAl, then used to construct single gene trees using IQ-TREE^73^, and visualized using FigTree. Tree outputs were manually screened for paralogs and contaminants. Cleaned gene trees missing over 40% analyzed genes were excluded and the remainder (Supplementary Table S3) were aligned using MAFFT L-INS-i again and trimmed once more with trimAl with the -automated1 option. SCaFoS^74^ was used to filter taxa and concatenate the resulting alignment consisting of 85 taxa, 190 proteins, and 45,976 amino acid sites. A final maximum likelihood phylogenomic tree was made using this alignment with IQ-TREE^73,75^ under the LG + C60 + F + G mixture model and 1000 UFB. To generate a rapid approximation of the profile mixture model for evaluating node support of crustacean gregarines a Posterior Mean-Site Frequency (PMSF) model^76^ was run on IQ-TREE using the initial maximum likelihood tree as the input guide tree and LG + C60 + F + G model with 200 non-parametric bootstrap replicates.

## Supplementary Information

Supplementary Information 1.

Supplementary Video 1.

Supplementary Video 2.

Supplementary Video 3.

Supplementary Video 4.

Supplementary Video 5.

Supplementary Video 6.

## Data Availability

Raw transcriptome reads are available on NCBI SRA under BioProject PRJNA1195655 and rRNA gene sequences are available on Genbank under accessions PQ738206-PQ738238. Transcriptome assemblies, predicted proteomes, alignments used to generate phylogenetic and phylogenomic trees, and host COI and SSU rRNA gene sequences are available on Mendeley Data under the 10.17632/vpsnhrkw3y.1
