# Supplementary material for: Phylogenomics reveals multiple evolutionary lineages of marine crustacean-infecting gregarine apicomplexans
Source: Sci Rep. 2026 Apr 20;16:18491. doi: 10.1038/s41598-026-46824-z (PMC13265913; doi:10.1038/s41598-026-46824-z)
Supplement: Supplementary file 1 — Supplementary Information 1. [file 41598_2026_46824_MOESM1_ESM.docx]

**Phylogenomic Diversity of Marine Crustacean-Infecting Gregarine Apicomplexans Within and Outside the Cephaloidophoroidea**

**Ina Na^1,^*, Victoria K. L. Jacko-Reynolds^1^, Corey C. Holt^1,2^, Patrick J. Keeling^1,^***

**Supplementary Figure S1**

Host images.(**A**) *Caprella* sp. (Host of *Cephaloidophora caprellina* n. sp.) (**B**) *Caprella* sp. (Host of *Cephaloidophora quadrae* n. sp.) (**C**) *Alienacanthomysis macropsis* (**D**) *Eriocheir sinensis* (**E**) *Ampithoe valida* (**F**) *Themisto* sp. (**G**) *Euphilomedes* sp.

**Supplementary Figure S2**

Fully detailed nuclear SSU+LSU rRNA gene phylogenetic tree representing major gregarine apicomplexan superfamilies generated using the GTR+F+R10 substitution model, 1000 UFBs, and 138 taxa. Bootstraps under 70% are omitted. Gregarines are highlighted with a dotted box. The tree is rooted on Dinoflagellates. Black circle = Nodes with full bootstrap support. Red bolded text = Newly added lineages. Brown text = Sequences from environmental or microbiome studies.

**Supplementary Figure S3**

Nuclear SSU+LSU rRNA gene phylogeny representing major groups on the tree of Apicomplexa. The maximum likelihood tree was generated using the GTR+F+R10 substitution model using 1000 UFB and 164 taxa. Nodes with full bootstrap support are indicated by a black circle and bootstraps under 70% are omitted.

**Supplementary Video 1. *Cephaloidophora quadrae* n. sp.** Bending and twisting movements of the *C. quadrae* primite while gliding.

**Supplementary Video 2. *Cephaloidophora cola* n. sp.** Undulating movements at the anterior portion of a *C. cola* cell.

**Supplementary Video 3. *Thiriotia zilla* n. sp.** Bending, twisting, and curling exhibited by *T. zilla* while gliding.

**Supplementary Video 4. *Thiriotia zilla* n. sp.** Gliding motility of *T. zilla*.

**Supplementary Video 5. *Ganymedes themistos*.** Contracting movement of the *G. themistos* epimerite.

**Supplementary Video 6. *Ganymedes themistos.*** Cells of *G. themistos* adhering to a glass slide while attempting to be pushed off by water movement generated via a glass pipette, then being physically pushed off the slide by the glass pipette tip.

**Supplementary Table S1:** Sample and Host Information.

| **Genbank Accession** | **isolate** | **Species** | **Date (MM-DD-YYYY)** | **Location and Method** | **Host** | **Host common name** | **Host Family** | **Host Order** | **Host Ecological Niche** | **Host COI Mendeley Data Filename** | **Host SSU Mendeley Data Filename** | **Geographic Coordinates** |
| --- | --- | --- | --- | --- | --- | --- | --- | --- | --- | --- | --- | --- |
| PQ738229 | **NaQua64** | Lentusidium euphilomedae | 9-12-2020 | Quadra. Hyacinthe Bay. Intertidal zone. Benthic light trap. | Euphilomedes sp. | seed shrimp | Philomedidae | Ostracoda | Benthos | N/A | Euphilomedes sp 1 SSU NaQua64 host.fasta | 50°06'54.4"N 125°13'13.9"W |
| PQ738230 | **NaQua66** | Lentusidium euphilomedae | 9-12-2020 | Quadra. Hyacinthe Bay. Intertidal zone. Benthic light trap. | Euphilomedes sp. | seed shrimp | Philomedidae | Ostracoda | Benthos | N/A | Euphilomedes sp 1 SSU NaQua66 host.fasta | 50°06'54.4"N 125°13'13.9"W |
| PQ738231 | **NaQua77** | Lentusidium euphilomedae | 9-13-2020 | Quadra. Hyacinthe Bay. Intertidal zone. Benthic light trap. | Euphilomedes sp. | seed shrimp | Philomedidae | Ostracoda | Benthos | N/A | Euphilomedes sp 1 SSU NaQua77 host.fasta | 50°06'54.4"N 125°13'13.9"W |
| PQ738209 | **NaQua166** | Cephaloidophora quadrae | 07-23-2021 | Quadra. Hyacinthe Bay. Intertidal zone. Benthic light trap. | Caprella sp. | skeleton shrimp | Caprellidae | Amphipoda | Benthos | N/A | Caprella sp 1 SSU NaQua166 host.fasta | 50°06'58.1"N 125°13'17.1"W |
| PQ738206 | **NaQua185** | Cephaloidophora alienae | 08-07-2021 | Quadra. Hyacinthe Bay. Intertidal zone. Benthic light trap. | Alienacanthomysis macropsis | mysid/opossum shrimp | Mysidae | Mysidacea | Benthos | N/A | Alienacanthomystis macropsis SSU NaQua185 host.fasta | 50°06'58.4"N 125°13'16.8"W |
| PQ738207 | **NaQua173** | Cephaloidophora caprellina | 08-06-2021 | Quadra Heroit Bay. Benthic light trap | Caprella sp. | skeleton shrimp | Caprellidae | Amphipoda | Benthos | N/A | Caprella sp 2 SSU NaQua173 host.fasta | 50°06'10.5"N 125°12'41.1"W |
| PQ738208 | **NaQua174** | Cephaloidophora caprellina | 08-06-2021 | Quadra Heroit Bay light trap | Caprella sp. | skeleton shrimp | Caprellidae | Amphipoda | Benthos | N/A | Caprella sp 2 SSU NaQua174 host.fasta | 50°06'10.5"N 125°12'41.1"W |
| PQ738210 | **NaQua179** | Cephaloidophora cola | 08-07-2021 | Quadra Heroit Bay. Intertidal zone. Benthic light trap. | Eriocheir sinensis | chinese mitten crab (juvenile) | Varunidae | Decapoda | Benthos | N/A | Eriocheir siensis SSU NaQua179 host.fasta | 50°06'10.5"N 125°12'41.1"W |
| PQ738211 | **NaQua180** | Cephaloidophora cola | 08-07-2021 | Quadra Heroit Bay Intertidal zone. Benthic light trap. | Eriocheir sinensis | chinese mitten crab (juvenile) | Varunidae | Decapoda | Benthos | N/A | Eriocheir sinensis SSU NaQua180 host.fasta | 50°06'10.5"N 125°12'41.1"W |
| PQ738232 | **NaQua183** | Thiriotia ampithae | 08-07-2021 | Quadra. Hyacinthe Bay. Intertidal zone. Benthic light trap. | Ampithoe valida | amphipod | Amphithoidae | Amphipoda | Benthos | Ampithoe valida COI NaQua183 host.fasta | N/A | 50°06'58.4"N 125°13'16.8"W |
| PQ738233 | **NaQua184** | Thiriotia ampithae | 08-07-2021 | Quadra. Hyacinthe Bay. Intertidal zone. Benthic light trap. | Ampithoe valida | amphipod | Amphithoidae | Amphipoda | Benthos | Ampithoe valida COI NaQua184 host.fasta | N/A | 50°06'58.4"N 125°13'16.8"W |
| PQ738219 | **NaQua215** | Ganymedes balani | 09-15-2021 | Beach near Quadra Institute Lab | Balanus glandula | acorn barnacle | Balanidae | Balanomorpha | Benthos | Balanus glandula SSU NaQua215 host.fasta | N/A | 50°06'56.6"N 125°13'16.4"W |
| PQ738221 | **NaQua218** | Ganymedes balani | 09-15-2021 | Beach near Quadra Institute Lab | Balanus glandula | acorn barnacle | Balanidae | Balanomorpha | Benthos | Balanus glandula COI NaQua218 host.fasta | N/A | 50°06'56.6"N 125°13'16.4"W |
| PQ738220 | **NaQua216** | Ganymedes balani | 09-15-2021 | Beach near Quadra Institute Lab | Balanus glandula | acorn barnacle | Balanidae | Balanomorpha | Benthos | Balanus glandula COI NaQua216 host.fasta | N/A | 50°06'56.6"N 125°13'16.4"W |
| PQ738212 | **NaQua216b** | Cephaloidophora hippola | 09-15-2021 | Beach near Quadra Institute Lab | Hippolyte sp. | shrimp | Hippolytidae | Decapoda | Benthos | N/A | Hippolyte sp 1 SSU NaQua216b host.fasta | 50°06'56.6"N 125°13'16.4"W |
| PQ738216 | **NaQua221** | Cephaloidophora squarepantsi | 09-15-2021 | Quadra. Hyacinthe Bay. Intertidal zone. Benthic light trap. | Neomysis sp. | mysid/opossum shrimp | Mysidae | Mysidacea | Planktonic/Benthos | N/A | Neomysis sp 1 SSU NaVic221 host.fasta | 50°06'57.8"N 125°13'17.3"W |
| PQ738217 | **NaQua222** | Cephaloidophora squarepantsi | 09-15-2021 | Quadra. Hyacinthe Bay. Intertidal zone. Benthic light trap. | Neomysis sp. | mysid/opossum shrimp | Mysidae | Mysidacea | Planktonic/Benthos | N/A | Neomysis sp 1 SSU NaVic222 host.fasta | 50°06'57.8"N 125°13'17.3"W |
| PQ738218 | **NaQua223** | Cephaloidophora squarepantsi | 09-15-2021 | Quadra. Hyacinthe Bay. Intertidal zone. Benthic light trap. | Neomysis sp. | mysid/opossum shrimp | Mysidae | Mysidacea | Planktonic/Benthos | N/A | Neomysis sp 1 SSU NaVic223 host.fasta | 50°06'57.8"N 125°13'17.3"W |
| PQ738213 | **NaQua3084** | Cephaloidophora squarepantsi | 11-2-2021 | Quadra. Hyacinthe Bay. Intertidal zone. Benthic light trap. | Neomysis sp. | mysid/opossum shrimp | Mysidae | Mysidacea | Planktonic/Benthos | N/A | Neomysis sp 1 SSU NaQua3084 host.fasta | 50°06'57.8"N 125°13'17.3"W |
| PQ738214 | **NaQua3092** | Cephaloidophora squarepantsi | 11-2-2021 | Quadra. Hyacinthe Bay. Intertidal zone. Benthic light trap. | Neomysis sp. | mysid/opossum shrimp | Mysidae | Mysidacea | Planktonic/Benthos | N/A | Neomysis sp 1 SSU NaQua3092 host.fasta | 50°06'57.8"N 125°13'17.3"W |
| PQ738215 | **NaQua3099** | Cephaloidophora squarepantsi | 11-2-2021 | Quadra. Hyacinthe Bay. Intertidal zone. Benthic light trap. | Neomysis sp. | mysid/opossum shrimp | Mysidae | Mysidacea | Planktonic/Benthos | N/A | Neomysis sp 1 SSU NaQua3099 host.fasta | 50°06'57.8"N 125°13'17.3"W |
| PQ738234 | **NaQua3397** | Thiriotia cypherae | 11-3-2021 | deep sea plankton tow, 240m depth, quadra island | Cyphocaris challengeri | amphipod | Cyphocarididae | Amphipoda | Planktonic | N/A | Cyphocaris challengeri SSU NaQua3397 host.fasta | 50°06'57.8"N 125°13'17.3"W |
| PQ738235 | **NaQua3477** | Thiriotia cypherae | 11-4-2021 | deep sea plankton tow, 240m depth, quadra island | Cyphocaris challengeri | amphipod | Cyphocarididae | Amphipoda | Planktonic | N/A | Cyphocaris challengeri SSU NaQua3477 host.fasta | 50°06'57.8"N 125°13'17.3"W |
| PQ738236 | **NaQua3490** | Thiriotia cypherae | 11-4-2021 | deep sea plankton tow, 240m depth, quadra island | Cyphocaris challengeri | amphipod | Cyphocarididae | Amphipoda | Planktonic | N/A | Cyphocaris challengeri SSU NaQua3490 host.fasta | 50°06'57.8"N 125°13'17.3"W |
| PQ738237 | **NaQuaGreg1** | Thiriotia cypherae | 11-4-2021 | deep sea plankton tow, 240m depth, quadra island | Cyphocaris challengeri | amphipod | Cyphocarididae | Amphipoda | Planktonic | N/A | N/A | 50°06'57.8"N 125°13'17.3"W |
| PQ738238 | **NaVict234** | Thiriotia zilla | 02-07-2022 | Intertidal seagrass zone. Beach/Dock area. Clover Point, Victoria. | Scyra sp. | sharp-nosed crab (juvenile) | Epialtidae | Decapoda | Benthos | Scyra sp COI NaVic234 host.fasta | N/A | 48°24'10.0"N 123°20'54.6"W |
| PQ738222 | **NaGal259** | Ganymedes themistos | 02-28-2022 | Galiano light trap | Themisto libellula | amphipod | Hyperiidae | Amphipoda | Planktonic/Benthos | N/A | Themisto sp SSU NaGal259 host.fasta | 48°59'40.7"N 123°35'04.2"W |
| PQ738223 | **NaGal260** | Ganymedes themistos | 02-28-2022 | Galiano light trap | Themisto libellula | amphipod | Hyperiidae | Amphipoda | Planktonic/Benthos | N/A | Themisto sp SSU NaGal260 host.fasta | 48°59'40.7"N 123°35'04.2"W |
| PQ738224 | **NaGal261** | Ganymedes themistos | 02-28-2022 | Galiano light trap | Themisto libellula | amphipod | Hyperiidae | Amphipoda | Planktonic/Benthos | Themisto sp COI NaGal261 host.fasta | N/A | 48°59'40.7"N 123°35'04.2"W |
| PQ738225 | **NaGal262** | Ganymedes themistos | 02-28-2022 | Galiano light trap | Themisto libellula | amphipod | Hyperiidae | Amphipoda | Planktonic/Benthos | Themisto sp COI NaGal262 host.fasta | N/A | 48°59'40.7"N 123°35'04.2"W |
| PQ738226 | **NaGal263** | Ganymedes themistos | 02-28-2022 | Galiano light trap | Themisto libellula | amphipod | Hyperiidae | Amphipoda | Planktonic/Benthos | Themisto sp COI NaGal263 host.fasta | N/A | 48°59'40.7"N 123°35'04.2"W |
| PQ738227 | **NaGal264** | Ganymedes themistos | 02-28-2022 | Galiano light trap | Themisto libellula | amphipod | Hyperiidae | Amphipoda | Planktonic/Benthos | N/A | Themisto sp SSU NaGal264 host.fasta | 48°59'40.7"N 123°35'04.2"W |
| PQ738228 | **NaGal265** | Ganymedes themistos | 02-28-2022 | Galiano light trap | Themisto libellula | amphipod | Hyperiidae | Amphipoda | Planktonic/Benthos | Themisto sp COI NaGal265 host.fasta | N/A | 48°59'40.7"N 123°35'04.2"W |

**Supplementary Table S2:** Measurements and motility of novel crustacean-infecting gregarines and *Ganymedes themistos*

| **Species** | **N=** | **Nucleus diameter (µm)** | **Length (µm)** | **Width**  **(µm)** | **Type** | **Septum or septum-like indentation** | **Demarcation Separating the Ectoplasm and Endoplasm** | **Motility** |
| --- | --- | --- | --- | --- | --- | --- | --- | --- |
| *Cephaloidophora hippola* n. sp. | 2 | 11 | 30 | 21 | gamont (primite) | septum | yes | not observed |
|  |  | 12 | 37 | 23 | gamont (satellite) |  |  |  |
|  | *average* | *11.5* | *33.5* | *22* |  |  |  |  |
| *Cephaloidophora caprellina* n. sp. | 3 | not observed | 72 | 49 | trophozoite | septum | yes | not observed |
|  |  | not observed | 83 | 51 | gamont (primite) |  |  |  |
|  |  | not observed | 52 | 27 | gamont (satellite) |  |  |  |
|  | *average* | *n/a* | *69* | *42.333333* |  |  |  |  |
| *Cephaloidophora alienae* n. sp. | 2 | not observed | 42 | 40 | gamont (primite) | septum | yes | pulsing in anterior direction |
|  |  | 13 | 40 | 39 | gamont (satellite) |  |  |  |
|  | *average* | *13* | *41* | *39.5* |  |  |  |  |
| *Cephaloidophora quadrae* n. sp. | 2 | 6 | 20 | 10 | gamont (primite) | septum | yes | gliding in anterior direction, bending and twisting/constricting movements |
|  |  | not observed | 11 | 5 | gamont (satellite) |  |  |  |
|  | *average* | *6* | *15.5* | *7.5* |  |  |  |  |
| *Cephaloidophora cola* n. sp. | 2 | 12 | 110 | 32 | trophozoite | septum | yes | slight undulating movement in cell anterior direction, otherwise no other motility |
|  |  | 10 | 92 | 15 | trophozoite |  |  |  |
|  | *average* | *11* | *101* | *23.5* |  |  |  |  |
| *Cephaloidophora squarepantsi* n. sp. | 8 | 20 | 80 | 71 | trophozoite | septum | yes | pulsing in anterior direction |
|  |  | 30 | 97 | 90 | trophozoite |  |  |  |
|  |  | 20 | 96 | 89 | trophozoite |  |  |  |
|  |  | 16 | 82 | 73 | trophozoite |  |  |  |
|  |  | 30 | 99 | 92 | trophozoite |  |  |  |
|  |  | 26 | 80 | 72 | trophozoite |  |  |  |
|  |  | 26 | 96 | 88 | trophozoite |  |  |  |
|  |  | 18 | 84 | 75 | trophozoite |  |  |  |
|  | *average* | *23.25* | *89.25* | *81.25* |  |  |  |  |
| *Thiriotia ampithae* n. sp. | 2 | 6 | 50 | 9 | trophozoite | not observed | not observed | not observed |
|  |  | 5 | 57 | 11 | trophozoite |  |  |  |
|  | *average* | *5.5* | *53.5* | *10* |  |  |  |  |
| *Thiriotia cypherae* n. sp. | 2 | 32 | 271 | 45 | gamont (primite) | not observed | yes | gliding in anterior direction |
|  |  | 20 | 213 | 32 | gamont (satellite) |  |  |  |
|  | *average* | *26* | 242 | 38.5 |  |  |  |  |
|  | 2 | 21 | 240 | 33 | trophozoite |  |  |  |
|  |  | 22 | 214 | 42 | trophozoite |  |  |  |
|  | *average* | *21.5* | *227* | *37.5* |  |  |  |  |
| *Thiriotia zilla* n. sp. | 5 | 40 | 1820 | 150 | trophozoite | not observed | not observed | bending, twisting, curling as well as gliding quickly in anterior direction |
|  |  | 44 | 1920 | 150 | trophozoite |  |  |  |
|  |  | 60 | 2005 | 160 | trophozoite |  |  |  |
|  |  | 52 | 1985 | 160 | trophozoite |  |  |  |
|  |  | 42 | 1655 | 150 | trophozoite |  |  |  |
|  | *average* | *47.6* | *1877* | *154* |  |  |  |  |
| *Ganymedes balani* n. sp. | 4 type A | 12 | 62 | 30 | gamont (primite) | septum-like indentation | yes | gliding in anterior direction |
|  |  | 13 | 68 | 24 | gamont (satellite) |  |  |  |
|  |  | 12 | 62 | 24 | gamont (primite) |  |  |  |
|  |  | 12 | 70 | 26 | gamont (satellite) |  |  |  |
|  | *average* | *12.25* | *65.5* | *26* |  |  |  |  |
|  | 6 type B | 10 | 60 | 20 | gamont (primite) |  |  |  |
|  |  | 8 | 50 | 20 | gamont (satellite) |  |  |  |
|  |  | 10 | 52 | 22 | gamont (primite) |  |  |  |
|  |  | 9 | 50 | 20 | gamont (satellite) |  |  |  |
|  |  | 8 | 50 | 20 | gamont (primite) |  |  |  |
|  |  | 9 | 46 | 20 | gamont (satellite) |  |  |  |
|  | *average* | *9* | *51.333333* | *20.333333* |  |  |  |  |
| *Ganymedes themistos* | 10 | 38 | 410 | 50 | gamont (primite) | septum-like indentation | yes | gliding in anterior direction |
|  |  | 40 | 180 | 60 | gamont (satellite) |  |  |  |
|  |  | 32 | 370 | 50 | gamont (primite) |  |  |  |
|  |  | 30 | 170 | 50 | gamont (satellite) |  |  |  |
|  |  | 32 | 340 | 40 | gamont (primite) |  |  |  |
|  |  | 24 | 150 | 50 | gamont (satellite) |  |  |  |
|  |  | 28 | 410 | 60 | gamont (primite) |  |  |  |
|  |  | 20 | 190 | 60 | gamont (satellite) |  |  |  |
|  |  | 30 | 320 | 50 | gamont (primite) |  |  |  |
|  |  | 30 | 150 | 60 | gamont (satellite) |  |  |  |
|  | *average* | *30.4* | *269* | *53* |  |  |  |  |
| *Lentusidium euphilomedae* n. gen. et sp. | 10 | 30 | 280 | 125 | trophozoite | not observed | not observed | gliding in anterior direction |
|  |  | 40 | 150 | 80 | trophozoite |  |  |  |
|  |  | 40 | 210 | 130 | trophozoite |  |  |  |
|  |  | 40 | 160 | 80 | trophozoite |  |  |  |
|  |  | 38 | 150 | 95 | trophozoite |  |  |  |
|  |  | 32 | 150 | 60 | trophozoite |  |  |  |
|  |  | 44 | 200 | 80 | trophozoite |  |  |  |
|  |  | 42 | 110 | 80 | trophozoite |  |  |  |
|  |  | 22 | 65 | 50 | trophozoite |  |  |  |
|  |  | 26 | 100 | 60 | trophozoite |  |  |  |
|  | *average* | *35.4* | *157.5* | *84* |  |  |  |  |

**Supplementary Table S3:** Genes used for multi-gene phylogenetic analyses

| **Orthologs** |
| --- |
| rpl7a |
| eef2 |
| rpl3 |
| rpl5 |
| rplp0 |
| vpc |
| rps4y1 |
| rpl18 |
| rpsa |
| tubb |
| rpl9 |
| rps9 |
| sec61 |
| rps12 |
| tcp1-delta |
| RPS24 |
| atp6v1b |
| bat1 |
| rpl13 |
| sars |
| RUVBL1 |
| psmb3 |
| rps3 |
| rps6 |
| tcp1-gamma |
| tcp1-zeta |
| KARS |
| ap1m1 |
| eif5A |
| rpl12 |
| rpl13a |
| rpl19 |
| RPS19 |
| atp6v1d |
| ran |
| rpl4 |
| rpl7 |
| rps5 |
| tcp1-alpha |
| tcp1-epsilon |
| EIF1B |
| KRR1 |
| atp6v1a |
| eif2g |
| nop56 |
| nsf |
| psma2 |
| psma5 |
| rpl10 |
| rpl11 |
| rpl8 |
| tcp1-theta |
| erf3b |
| psma6 |
| psmb2 |
| psmb5 |
| psmb6 |
| psmb7 |
| psmc1 |
| psmc6 |
| rpl24 |
| rps10 |
| rps11 |
| MAT1A |
| PCNA |
| VPS26B |
| eif2b |
| eif6 |
| gdi2 |
| pno1 |
| psma3 |
| psmc2 |
| rpl32 |
| rps15a |
| rps2 |
| rps8 |
| BYSL |
| DKC1 |
| GNL2 |
| calm |
| eif5b |
| fbl |
| mcm2 |
| psma4 |
| EIF3I |
| atp6v1e |
| etf1 |
| gnb2L1 |
| mcm7 |
| nop58 |
| psma7 |
| psmd14 |
| rpl36a |
| ASF1 |
| DRG2 |
| NSA2 |
| atp6v1c |
| eif1a |
| mcm5 |
| nhp2 |
| psmc3 |
| psmd1 |
| rpl14 |
| rpl15 |
| rps16 |
| rps26 |
| EIF2A |
| POLR2F |
| RFC2 |
| psma1 |
| psmc4 |
| psmc5 |
| rpl30 |
| NIP7 |
| PSMD12 |
| RPL34 |
| psmb1 |
| rad51 |
| rpl17 |
| rps17 |
| rps18 |
| rps3a |
| IMP4 |
| NCBP2 |
| NOP2 |
| POLR2B |
| YKT6 |
| crfg |
| mcm3 |
| rpl10a |
| rpl18a |
| rpl21 |
| tcp1-beta |
| ABCE |
| COP-beta |
| GRWD1 |
| emg1 |
| gpn1 |
| FCF1 |
| nhp2L1 |
| rps13 |
| rps23 |
| tcp1-eta |
| AGX |
| DIMT1L |
| POLR2H |
| RBM19 |
| RFC4 |
| RFC5 |
| hsp90 |
| psmb4 |
| suclg1 |
| COPG2 |
| MAK16 |
| DPH1 |
| XPO1 |
| mcm6 |
| rpl6 |
| gpn3 |
| mcm4 |
| rpl35 |
| rps14 |
| xpb |
| POLR1C |
| RCL1 |
| RPF1 |
| metap2 |
| rps15 |
| rps20 |
| tbp |
| DCAF13 |
| FTSJ1 |
| gpn2 |
| srp54 |
| CHURC1FNTB |
| LSM4 |
| PRPF8 |
| SND1 |
| eftud2 |
| rpl27a |
| rpl31 |
| tubg |
| AP4S1 |
| KPNB1 |
| NAT10 |
| AP1S2 |
| METTL1 |
| RPS25 |
| rpl26 |
| vps4 |
